# Cysteine-to-lysine transfer antibody fragment conjugation†
†Electronic supplementary information (ESI) available. See DOI: 10.1039/c9sc03825f


**DOI:** 10.1039/c9sc03825f

**Published:** 2019-10-11

**Authors:** Nafsika Forte, Irene Benni, Kersti Karu, Vijay Chudasama, James R. Baker

**Affiliations:** a Department of Chemistry , University College London , 20 Gordon Street , London , WC1H 0AJ , UK . Email: j.r.baker@ucl.ac.uk ; Email: v.chudasama@ucl.ac.uk; b Research Institute for Medicines (iMed.ULisboa) , Faculty of Pharmacy , Universidade de Lisboa , Lisbon , Portugal

## Abstract

The modification of lysine residues with acylating agents has represented a ubiquitous approach to the construction of antibody conjugates, with the resulting amide bonds being robustly stable and clinically validated. However, the conjugates are highly heterogeneous, due to the presence of numerous lysines on the surface of the protein, and greater control of the sites of conjugation are keenly sought. Here we present a novel approach to achieve the targeted modification of lysines distal to an antibody fragment's binding site, using a disulfide bond as a temporary ‘hook’ to deliver the acylating agent. This cysteine-to-lysine transfer (CLT) methodology offers greatly improved homogeneity of lysine conjugates, whilst retaining the advantages offered by the formation of amide linkages.

## Abstract 2

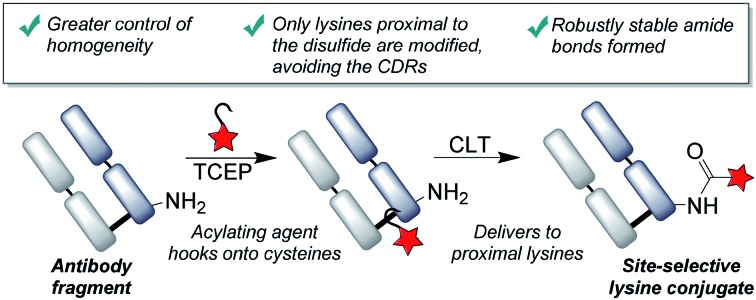
Site-selective antibody fragment conjugation is achieved by using a disulfide bond to ‘hook’ and deliver thioester acylating agents to specific lysines.

## Introduction

Over the past decades, antibody bioconjugation has emerged as a powerful tool, providing new avenues for the development of therapeutics and diagnostics.^1^ Combining the exquisite targeting ability of antibodies with small molecules has enabled access to a broad range of constructs, including antibody–drug conjugates,^2^ bispecifics,^3^ radioimmunoconjugates,^4^ as well as antibody–nanoparticle conjugates^5^ and targeted imaging agents.^6^ Notably, antibody fragments have shown distinct advantages over full immunoglobulins, including enhanced tumour penetration, lower immunogenicity risk, accelerated renal clearance (tunable half-lives, *e.g.* by PEGylation) and production in cheaper prokaryotic expression systems.^7^–^9^

For the next generation of antibody conjugates, it has been demonstrated that site-selective modification strategies that afford robustly stable constructs are vital to ensure superior *in vivo* outcomes.^1^,^10^,^11^ The use of genetic engineering to incorporate cysteine mutants,^10^ unnatural amino-acids^12^ or enzymatically-recognised handles^13^ has enabled antibody modification with an unprecedented degree of site-selectivity. However, with these approaches, further input of resources in the antibody development phase is required and variable protein expression yields, disulfide scrambling or aggregation are limitations often witnessed.^10^,^14^–^16^

Alternatively, we and others have recently described the development of reagents which are able to modify native disulfide bonds by re-bridging the two cysteine residues, producing homogeneous antibody conjugates.^17^–^24^ Importantly, the structural integrity of the antibody is maintained, contrary to targeting each cysteine residue independently, which has been shown to reduce the stability of the antibody *in vivo*.^25^ Whilst disulfide bridging is a promising strategy, the resultant conjugates are yet to be validated in the clinic.

**Fig. 1:**
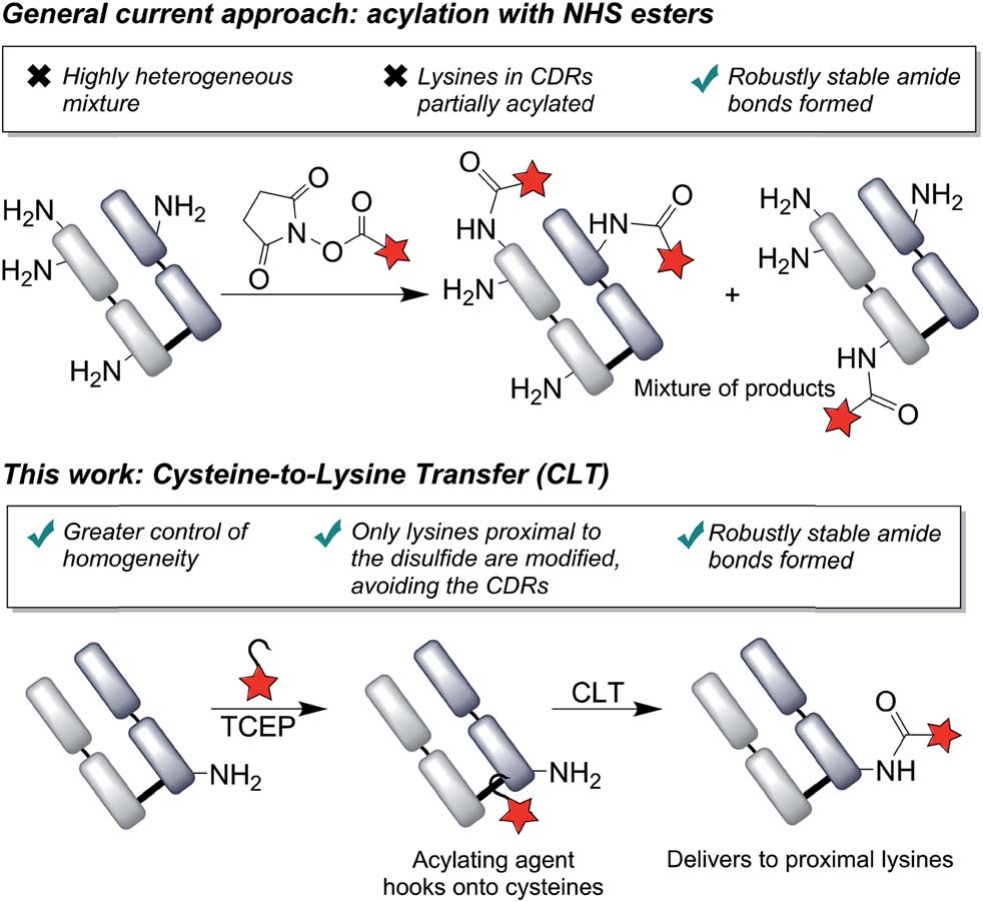
We describe the use of proximal cysteines to deliver acylation reagents to specific lysine residues in an antibody fragment, improving the homogeneity of these conjugates, whilst retaining the robustly stable amide linkages; CDRs = complementarity-determining regions.

Labeling *via* the primary amino groups on lysine residues has been heavily pursued, due to the advantages of using readily available acylating agents (*e.g.* NHS esters) to form robustly stable, clinically validated amide bonds.^26^ However, due to the multitude of surface accessible lysine residues, heterogeneous mixtures are inevitably obtained with batch-to-batch variability and unpredictable pharmacokinetic properties.^27^,^28^ An ideal approach to antibody modification would involve the site-selective labeling of lysines by acylation, as it would fulfil both the criteria of homogeneity and robust stability. Reagents have been described which offer greater selectivity for certain lysines than conventional reagents by exploiting subtle differences in p*K*_a_'s of lysines or local environments.^29^,^30^ We envisaged that an alternative approach to achieve selectivity would be to use proximal cysteine residues as ligating ‘hooks’, delivering acylating agents specifically to certain lysine residues (Fig. 1). This cysteine-to-lysine transfer (CLT) methodology would offer new opportunities in accessing site-selective protein conjugates more widely, building on recent reports of using reversible bonds to deliver reactive functional groups to specific amino-acids,^31^ including work by Bertozzi and coworkers on the use of nitrile reagents to modify specifically designed cysteine-containing peptides by S-to-N transfer to targeted lysine residues.^32^

## Results and discussion

**Fig. 2:**
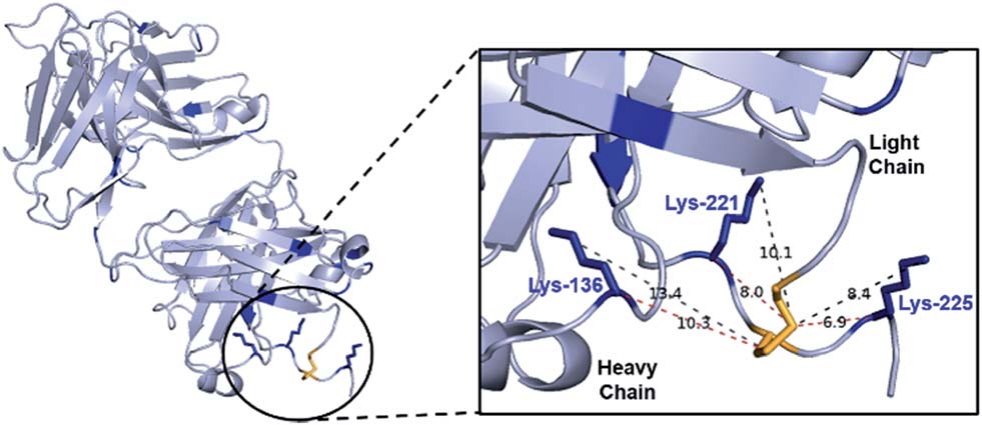
Fab structure, derived from PDB file 1HZH, human IgG against HIV-1. This has high structural similarity to trastuzumab Fab, which lacks several key amino acids D224–H227 in PDB; see Fig. S32† which shows mapped on structures. The distances (in Å) from the disulfide bond to either the nitrogen of the lysine or the α-carbon are shown as a guide to proximity, whilst recognizing the high flexibility in the system.

We chose to develop the CLT strategy for antibody conjugation on the Fab fragment of Her2-targeting breast cancer drug trastuzumab. Specifically, trastuzumab fragment conjugates are of widespread interest for drug delivery and imaging applications,^33^–^35^ and the presence of 26 lysines and a single disulfide bond would allow clear interpretation of the viability of the CLT strategy. Analysis of the region around the Fab disulfide revealed three proximal lysine residues on the heavy-chain (K136, K221, K225) and none on the light chain (Fig. 2).

Thioesters present an ideal reactivity profile to achieve the desired initial cysteine acylation and subsequent *S*,*N*-acyl transfer onto proximal lysines. They are >100 times more reactive than the corresponding oxo-esters to nucleophiles such as amines, whilst being less reactive to hydrolysis.^36^ This is exploited by nature, *e.g.* in acetoacetyl CoA in the Krebs cycle,^37^ in the ubiquitination of proteins,^38^ and in intein splicing.^39^ Native chemical ligation (NCL) uses this reactivity to achieve selective amine-acylation on peptides and proteins *via* an *S*,*N*-acyl transfer involving a 5-membered ring intermediate.^40^ This concept has also recently been applied to larger, macrocyclic intermediates,^41^ such as the use of internal cysteines in peptides to accelerate ligation to the N-terminus.^42^–^45^

**Fig. 3:**
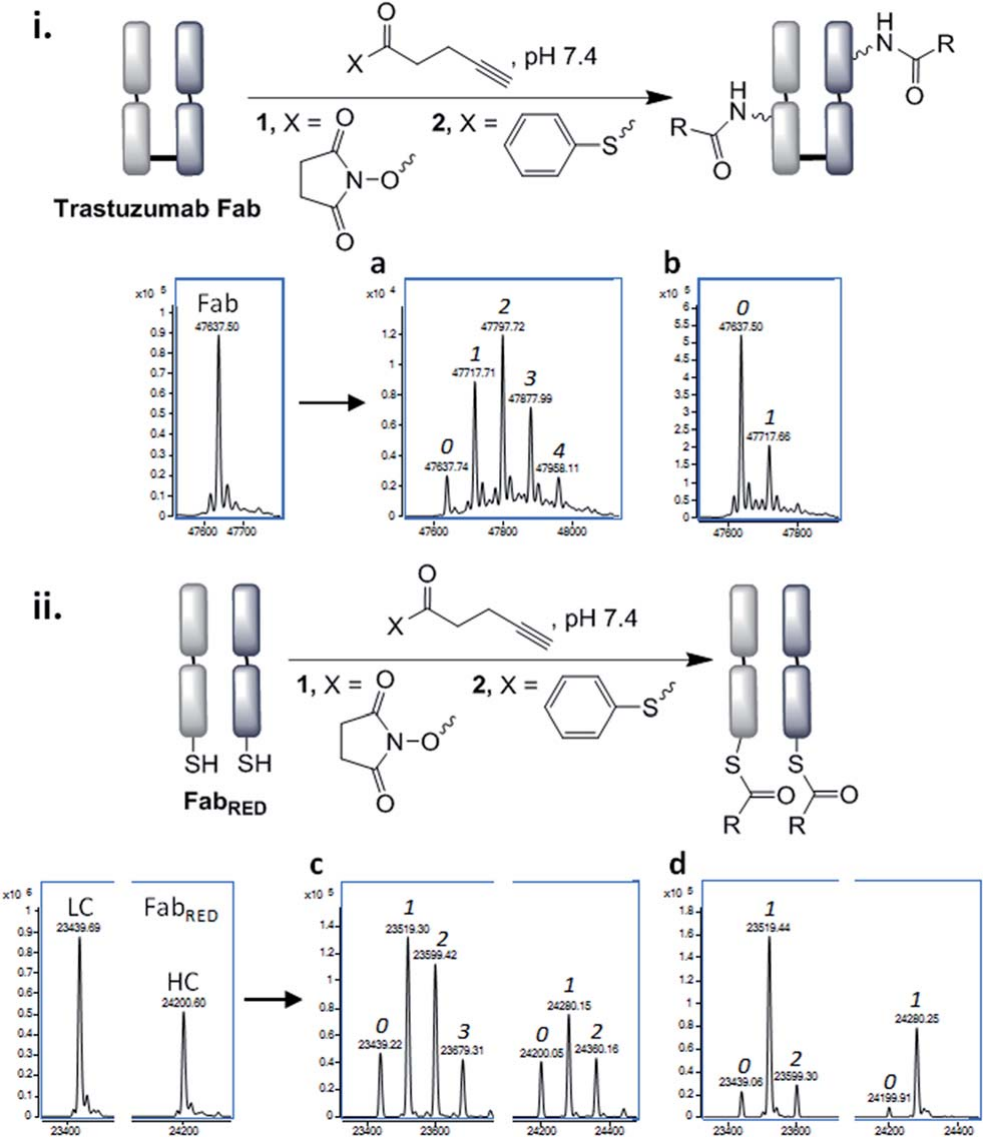
Acylation of trastuzumab Fab fragment; (i) in its native form with: (a) NHS ester **1** (2.5 eq., pH 7.4, 4 °C, 16 h), (b) arylthioester **2** (10 eq., pH 7.4, 15 min, RT), (ii) pre-reduced (with TCEP) with: (c) NHS ester **1** (2.5 eq., pH 7.4, 22 °C, 1 h), (d) arylthioester **2** (10 eq., pH 7.4, 15 min, RT); 0, 1, 2, 3, 4 refer to the number of acyl groups added per species.

In order for the CLT strategy to work, a thioester would need to react with the Fab fragment only upon reduction of the disulfide bond. Guided by NCL, which employs aryl thioesters to achieve efficient thiol–thioester exchange, we began our studies by treating Fab and reduced Fab with thiophenyl thioester **2** along with a common acylating agent NHS ester **1**. The number of acylations were identified by LC-MS (Fig. 3, see ESI† for raw LC-MS data). The NHS ester **1** generated a statistical distribution profile, consistent with non-selective lysine conjugation, upon reaction with either native or reduced Fab (Fig. 3a and c, respectively). The aryl thioester **2** demonstrated high selectivity for transthioesterification with reduced Fab, but the presence of double acylated light chain (LC) along with unmodified LC confirmed that a small amount of background lysine conjugation was occurring (Fig. 3d). This was further observed in the control reaction with native Fab, which showed a single acylation (Fig. 3b); treatment of this species with TCEP indicated that a reactive lysine(s) is present on the LC, which is consistent with literature reports (Fig. S6†).^29^,^30^

**Fig. 4:**
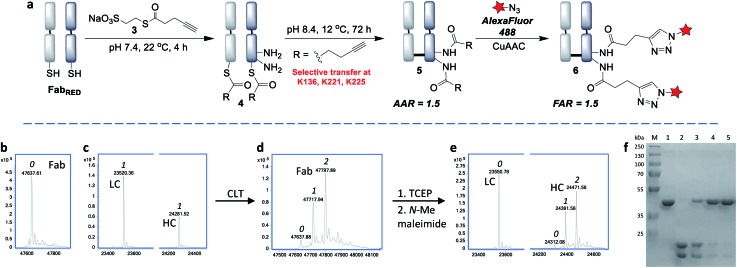
Cysteine-to-lysine transfer (CLT) strategy with MESNa thioester **3**: (a) general scheme, (b) LC-MS after reaction of native Fab with thioester **3**, (c) LC-MS after transthioesterification of reduced Fab with thioester **3**, (d) LC-MS of CLT conjugate **5**, (e) LC-MS of reduced CLT conjugate **5**, in which the cysteines have been capped with *N*-methylmaleimide (the masses include the expected addition of one *N*-methylmaleimide on the light chain and one on the heavy chain, see Fig. S15 for details†), (f) SDS-PAGE analysis: (M) molecular marker, (1) native Fab, (2) reduced Fab after transthioesterification with MESNa thioester **3**, (3) acyl transfer (24 h), (4) acyl transfer (48 h), (5) acyl transfer (72 h), *i.e.* CLT conjugate **5** (see Fig. S17† for complete SDS-PAGE analysis); 0, 1, 2 refer to the number of acyl groups added per species.

With the view to tuning down the reactivity of the thioester and hence avoid non-specific reactivity, we turned our attention to the use of alkyl thioesters. MESNa thioester **3** was found to be completely inert upon reaction with native Fab, even at 100 equiv. of reagent (Fig. 4b), while it was shown to undergo extremely selective transthioesterification with reduced Fab, affording solely the desired cysteinyl thioester conjugate **4** (Fig. 4c).^46^ Further confirmation that the cysteines were the sites of selective reaction was obtained by the addition of thiols (cysteine, 100 equiv.) post conjugation which readily cleaved the thioesters regenerating native Fab (Fig. S10†).

Having identified that alkyl thioesters can undergo selective transthioesterification, the next step was to attempt the *S*,*N*-acyl transfer to nearby lysine residues. While in NCL the initial transthioesterification is the rate determining step, in ligations proceeding *via* larger ring sizes, the *S*,*N*-acyl transfer becomes rate determining.^40^ Given the macrocyclic intermediates involved in the CLT strategy, we anticipated that this reaction would not be rapid and optimization of conditions to limit competing hydrolysis would be required. We analysed reactions up to 72 h and used LC-MS to reveal the alkyne : antibody ratios (AARs) (see ESI Table S2† for conditions examined). We observed that at physiological conditions (pH 7.4, RT), significant transfer had taken place, but the reaction was incomplete. Enticingly however, the major product formed was an acylated conjugate derived from the reoxidised native Fab, confirming that the cysteines had been liberated and had spontaneously reoxidised (see also SDS-PAGE, Fig. 4f), which negated the requirement for an extra oxidation step.

By increasing the temperature to 37 °C and pH to 8.0 the transfer reaction was pushed to completion in just 24 h. However, the AAR of 1.0 revealed that ∼50% of competing hydrolysis was taking place. To minimise this, we identified that lower temperature and higher pH gave the best yield of transfer. The final optimised conditions of 12 °C, pH 8.4, 72 h successfully afforded CLT conjugate **5** with an average AAR of 1.5 (Fig. 4d and f, lane 5). Upon treatment with thiols (100 equiv. cysteine, pH 8.4, 37 °C, 2 h), no change in the AAR was witnessed (Fig. S14†), confirming that a robustly stable acylated conjugate was obtained. Reduction of the conjugate with TCEP, followed by capping with *N*-methylmaleimide confirmed that the acylation had taken place exclusively on the heavy chain, which was consistent with a transfer mechanism occurring (Fig. 4e). The conjugate was then subjected to tryptic digestion, followed by LC-MS/MS analysis (see ESI†). 100% sequence coverage was obtained and the sites of modifications were identified as K136, K221 and K225, being in accordance with predictions based on the Fab crystal structure. Size-exclusion analysis of CLT conjugate **5** confirmed that no aggregation had taken place and ELISA showed full retention of binding activity (Fig. S26 and S27†). Finally, CuAAC was employed to conjugate AlexaFluor488 azide to generate a functional antibody conjugate **6**. The fluorophore-to-antibody ratio (FAR) was determined to be 1.5 by UV absorbance (Fig. S16†), which supported the loading obtained by LC-MS.

**Fig. 5:**
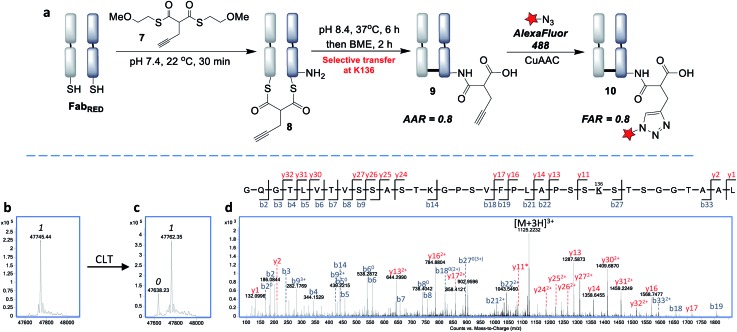
Site-selective cysteine-to-lysine transfer (CLT) with bis-thioester **7**: (a) general scheme, (b) LC-MS after transthioesterification of reduced Fab with thioester **7**, (c) LC-MS of CLT conjugate **9**, (d) LC-MS/MS of the Lys-136 modified peptide; 0 and 1 refer to the number of acyl groups added per species.

Next, we envisaged that the use of a bis-thioester would enable an alternative stoichiometry for this CLT strategy and postulated that the rigidity of a bridged system may further control the regioselectivity. We synthesised bis-thioester **7** using 2-methoxyethanethiol to infer water solubility, whilst avoiding purification issues associated with the highly polar bis-MESNa adducts. Treatment of reduced Fab with this reagent (100 equiv.) selectively afforded bridged conjugate **8** (Fig. 5b) in just 30 min at RT (no reaction was observed with unreduced Fab; see Fig. S18†). The increased rate of this double transthioesterification is consistent with the electron-withdrawing effects of the β-carbonyl. Following the successful formation of conjugate **8**, the *S*,*N*-acyl transfer was examined. Through optimization (see ESI†), we observed that the ideal conditions were 6 h at 37 °C, with LC-MS analysis revealing that the dominant product was the AAR 1 CLT conjugate **9**, in which the transfer was accompanied by hydrolysis of the second thioester. A further 2 h treatment with BME was identified as required to cleave off remaining traces of mono-thioesters which had formed due to competing hydrolysis, to afford a final AAR 0.8 conjugate (Fig. 5c). Incubation with BME also served to confirm the robust stability of CLT construct **9**, as no change in the AAR was witnessed. Following enzymatic digestion and LC-MS/MS analysis, we were pleased to find that this reaction was site-selective for K136 (Fig. 5d; 95% sequence coverage was obtained with 100% coverage of lysine residues). As only one lysine is reactive to acylation in this more constrained, bridged system, we infer that conformational effects must be playing a key role in driving this improved selectivity *i.e.* by holding a thioester in particular proximity of the K136 amino group. CLT conjugate **9** was also analysed by size exclusion chromatography, where no aggregation was observed to have taken place under the transfer conditions and ELISA analysis demonstrated full retention of binding activity (Fig. S26 and S27†). Subsequent AlexaFluor488 click conjugation generated fluorescent conjugate **10** with a matching FAR (Fig. S23†).

## Conclusion

In summary, cysteine-to-lysine transfer (CLT) methodology allows the construction of highly homogenous antibody fragment conjugates, whilst incorporating robustly stable, clinically validated amide linkages. The readily available thioester reagents are shown to react selectively with the cysteines obtained from the reduced interchain disulfide bond in a Fab, and then transfer at raised pH to specific proximal lysine residues, which are ideally placed distal from the binding site. By employing either mono- or bisthioesters we have shown it is possible to control the stoichiometry, to afford major products containing 2 or 1 acylations per disulfide. Whilst hydrolysis of the thioesters represents an expected competing background reaction, the efficiencies of the macrocyclic *S*,*N*-acyl transfers are impressively still 75–80%, and it is likely that these can be further tuned by reagent design in future generations of CLT reagents. Current site-specific approaches, for example using cysteine mutants,^47^ can achieve conjugation efficiencies over 90% and this should be the ultimate target. However, the use of an *S*,*N*-transfer reaction on a native protein to generate highly desirable amide linkages, with overall average loadings achieved of 1.5 and 0.8 per disulfide, already place this methodology in a suitable position for use in antibody conjugations and protein modifications more widely. It is also envisaged that CLT conjugation will allow the ready conversion of existing heterogeneous lysine reagents and conjugates to site-selective versions, building on the confidence in the resultant amide linkages whilst offering the prospect of improved therapeutic indexes and production processes known for homogeneous conjugates.

## Author contributions

N. F., V. C. and J. R. B. conceived and designed the project; N. F., V. C. and J. R. B. conceived and designed the chemistry and chemical biology experiments; N. F. and I. B. performed the chemistry and chemical biology experiments; K. K. performed the mass spectrometry experiments; N. F., V. C., K. K. and J. R. B. analysed the data; N. F., V. C. and J. R. B. co-wrote the paper.

## Conflicts of interest

The authors have filed a patent application on this technology with UCL Business.

## Supplementary Material

Supplementary information
