# Supplementary material for: Cysteine-to-lysine transfer antibody fragment conjugation
Source: Chem Sci. 2019 Oct 11;10(47):10919–24. doi: 10.1039/c9sc03825f (PMC7066670; doi:10.1039/c9sc03825f)
Supplement: Supplementary file 1 [file SC-010-C9SC03825F-s001.pdf]

Electronic Supplementary Material (ESI) for Chemical Science.
This journal is © The Royal Society of Chemistry 2019

Electronic Supplementary Information

Cysteine-To-Lysine Transfer Antibody Fragment Conjugation

Nafsika Forte,[†] *Irene Benni,*[†] *Kersti Karu,*[†] *Vijay Chudasama,*[†,‡,*] *and James R. Baker*[†,*]

† Department of Chemistry, University College London, 20 Gordon Street, London, WC1H 0AJ, UK

‡ Research Institute for Medicines (iMed.ULisboa), Faculty of Pharmacy, Universidade de Lisboa, Lisbon, Portugal

Table of Contents

Synthesis general remarks 4

Synthesis and characterization of compounds 5

- 2,5-dioxopyrrolidin-1-yl pent-4-ynoate (1)[1] 5
- S-phenyl pent-4-ynethioate (2) 7
- Sodium 2-(pent-4-ynoylthio)ethane-1-sulfonate (3) 8
- 2-(prop-2-yn-1-yl)malonic acid (11) 10
- S,S-bis(2-methoxyethyl) 2-(prop-2-yn-1-yl)propanebis(thioate) (7) 11

Bioconjugation general remarks 13

LC-MS general remarks 14

- Determination of Protein Masses by LC-MS 14
- Capillary LC-MS/MS analysis 15
- Database search 16

LC-MS analysis of native Fab and reduced Fab 17

Reaction of anti-HER2 Fab with NHS-ester alkyne 1 19

Reaction of reduced anti-HER2 Fab with NHS-ester alkyne 1 20

Reaction of anti-HER2 Fab with aryl thioester 2 21

Reaction of anti-HER2 Fab with aryl thioester 2, followed by reduction with TCEP 22

Reaction of reduced anti-HER2 Fab with aryl thioester 2 23

Reaction of anti-HER2 Fab with MESNa thioester 3 24

Transthioesterification of anti-HER2 Fab with MESNa thioester 3 (thioester conjugate 4) 25

Treatment of thioester conjugate 4 with cysteine 26

Optimized conditions for acyl transfer of thioester conjugate 4 at pH 8.4, 12 ^{o}C, 72 h (CLT conjugate 5) 27

Acyl transfer of thioester conjugate 4 under different pH/ temperature conditions 29

Treatment of CLT conjugate 5 with cysteine 32

TCEP reduction of CLT conjugate 5 and capping with *N*-methylmaleimide 33

CuAAC of CLT conjugate 5 with AlexaFluor488 to produce functionalized conjugate 6 35

SDS-PAGE analysis of cysteine-to-lysine transfer reaction employing MESNa thioester 3 36

Reaction of anti-HER2 Fab with bis-thioester 7 36

Transthioesterification of anti-HER2 Fab with bis-thioester 7 (bridged thioester conjugate 8) ... 37

Acyl transfer of bridged thioester conjugate 8 at pH 8.4, 12 ^{o}C, 48 h 39

Acyl transfer of bridged thioester conjugate 8 at pH 8.4, 37 ^oC, 6 h (CLT conjugate 9) 40
TCEP reduction of CLT conjugate 9 and capping with *N*-methylmaleimide.............................. 41
CuAAC of CLT conjugate 9 with AlexaFluor488 to produce functionalized conjugate 10 43
SDS-PAGE analysis of cysteine-to-lysine transfer reaction employing bis-thioester 7............... 45
Size Exclusion Chromatography (SEC).. 45
Enzyme-Linked Immunosorbent Assay (ELISA).. 46
Trypsin digestion of CLT conjugate 5 47
Chymotrypsin digestion of CLT conjugate 9.. 50
Overlaid Fab crystal structures derived from PDB files 1HZH and 6BAE 53
References.. 54

Synthesis general remarks

All chemical reagents and solvents were purchased from Sigma, AlfaAesar, Santa Cruz Biotechnology or VWR and used as received, without any further purification. All reactions were carried out at atmospheric pressure, under argon. Room temperature is defined as between 15-25 ^{o}C. The term *in vacuo* refers to solvent removal using Büchi rotary evaporation between 15-50°C, at approximately 10 mm Hg. Reactions were monitored by TLC, using TLC plates pre-coated with silica gel 60 F_{254} on aluminium (Merck KGaA). Detection was by UV (254 nm and 365 nm) or chemical stain ($KMnO_4$, ninhydrin, iodine). Column chromatography was carried out using a Biotage Isolera with GraceResolv™ silica flash cartridges. ^{1}H and ^{13}C NMR spectra were recorded at ambient temperature on a Bruker Advance AMX600 instrument, operating at 600 MHz for ^{1}H and at 150 MHz for ^{13}C in the stated solvent, using $CDCl_3$ (δ = 7.26) or CD_3OD (δ = 3.31) as the internal standard. Chemical shifts (δ) are reported in parts per million (ppm) and coupling constants (*J*) in Hertz (Hz). The multiplicity of each signal is indicated as s-singlet, d-doublet, t-triplet, q-quartet, quin-quintet, m-multiplet (i.e. complex peak obtained due to overlap) or a combination of these. All assignments were made with the aid of DEPT, COSY, HSQC, HMBC or NOESY correlation experiments. Infra-red spectra were recorded on a Bruker ALPHA FT-IR spectrometer operating in ATR mode, with frequencies given in reciprocal centimeters (cm^{-1}). The absorptions are characterized as s (sharp), br (broad), m (medium), w (weak). Melting points were taken on a Gallenkamp apparatus and are uncorrected. High and low resolution mass spectra were recorded on a VG70 SE mass spectrometer, operating in modes ESI, EI, or CI (+ or -) depending on the sample, at the Department of Chemistry, University College London or obtained by the EPSRC UK National Mass Spectrometry Facility (NMSF), Swansea.

Synthesis and characterization of compounds

2,5-dioxopyrrolidin-1-yl pent-4-ynoate (1)[1]

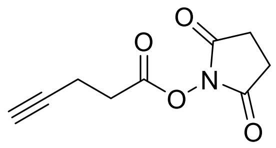

To 4-pentynoic acid (80.0 mg, 0.815 mmol) in CH_2Cl_2 (7 mL) was added EDC.HCl (234 mg, 1.22 mmol), followed by *N*-hydroxysuccinimide (140 mg, 1.22 mmol). The resultant mixture was stirred at RT for 16 h, under argon. The solvent was then removed *in vacuo* and purification by column chromatography (gradient elution from pet. ether to 50% EtOAc in pet. ether) afforded the target compound as a white solid (124 mg, 0.635 mmol, 78%).

mp 65-67 ^{o}C (lit. mp 70-72 ^{o}C)[1]; ^{1}H NMR (600 MHz, $CDCl_3$) δ_H 2.86 (t, 2H, *J* = 7.2, OC(O)C*H*$_2$), 2.81 (br s, 4H, 2 × NC(O)C*H*$_2$), 2.59 (td, 2H, *J* = 7.5, 2.6, CHCC*H*$_2$), 2.04 (1H, t, *J* = 2.7, C*H*); ^{13}C NMR (150 MHz, $CDCl_3$) δ_C 169.2 (N*C*(O)), 167.2 (O*C*(O)), 81.0 (*C*), 70.2 (*C*H), 30.4 (NC(O)*C*H$_2$), 25.7 (OC(O)*C*H$_2$), 14.2 (CHC*C*H$_2$); IR (solid) v_{max}/ cm^{-1} 3262 (m), 2955 (w), 2917 (w), 2849 (w), 1811 (m), 1786 (m), 1723 (s); LRMS (ES+) *m/z* 196 ([M+H]$^+$, 100), 176 (23), 149 (76), 132 (45), 116 (23); HRMS (ES+) calcd for $[C_{10}H_9NO_4]^+$ $[M+H]^+$ 196.0610, observed 196.0614.

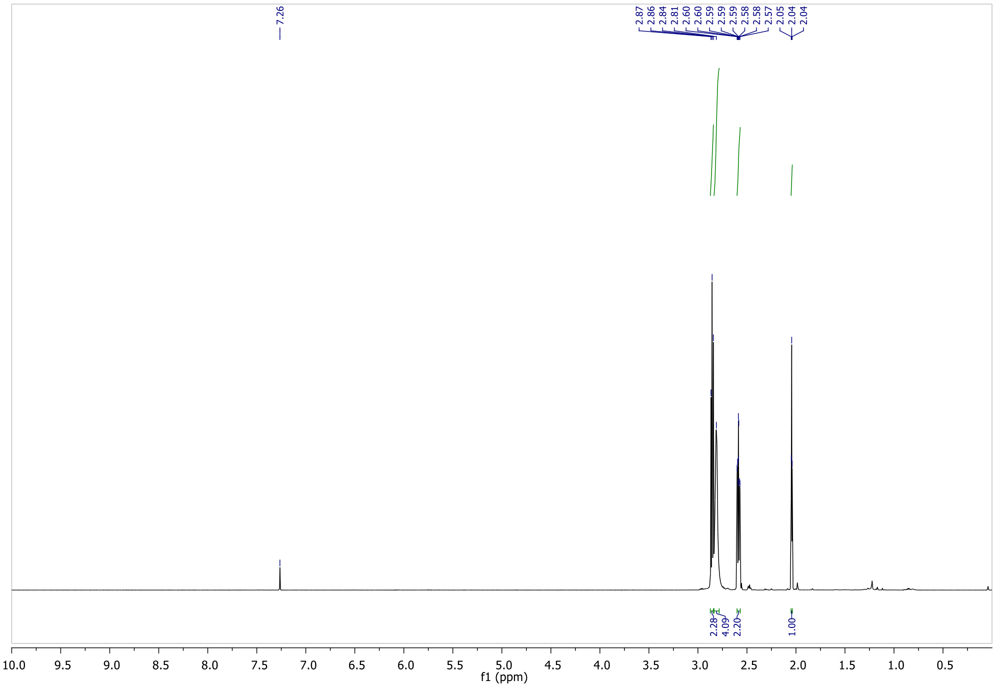
7.26
2.87
2.86
2.84
2.81
2.60
2.59
2.59
2.59
2.58
2.58
2.57
2.05
2.04
2.04
2.28
4.09
2.20
1.00
10.0
9.5
9.0
8.5
8.0
7.5
7.0
6.5
6.0
5.5
5.0
4.5
4.0
3.5
3.0
2.5
2.0
1.5
1.0
0.5
f1 (ppm)

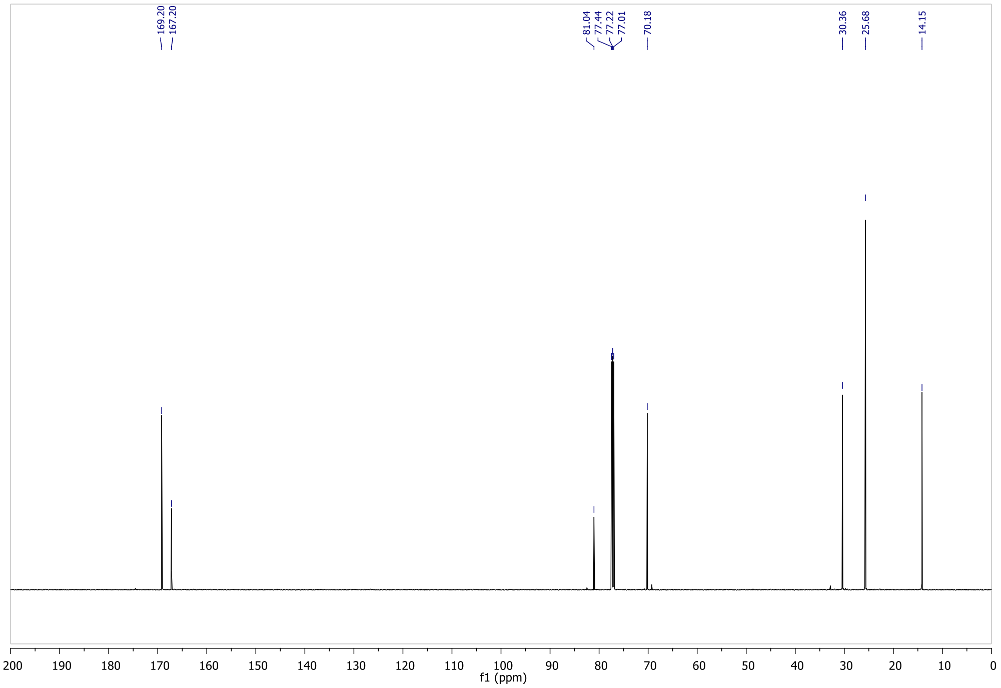
169.20
167.20
81.04
77.44
77.22
77.01
70.18
30.36
25.68
14.15
200
190
180
170
160
150
140
130
120
110
100
90
80
70
60
50
40
30
20
10
0
f1 (ppm)

S-phenyl pent-4-ynethioate (2)

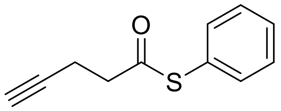

To 4-pentynoic acid (233 mg, 2.37 mmol) in dry THF (25 mL) was added thiophenol (162 µL, 1.58 mmol), followed by DMAP (10.0 mg, 0.0819 mmol) and EDC.HCl (455 mg, 2.37 mmol). The resultant mixture was stirred at RT for 16 h. The solvent was then removed *in vacuo* and EtOAc (10 mL) was added. The organic phase was washed with 10% aq. $Na_2S_2O_3$ (3 × 5 mL), 1M aq. HCl (3 × 5 mL), sat. aq. $NaHCO_3$ (3 × 5 mL), brine (3 × 5 mL), dried (Na_2SO_4) and concentrated. Purification by column chromatography (gradient elution from pet. ether to 10% EtOAc in pet. ether) afforded the target compound as a colourless oil (183 mg, 0.96 mmol, 61%).

^{1}H NMR (600 MHz, $CDCl_3$) δ_H 7.43-7.42 (br s, 5H, 5 × ArC*H*), 2.91 (t, 2H, *J* = 7.2, C(O)CH_2), 2.58 (td, 2H, *J* = 7.6, 2.7, C(O)CH_2CH_2), 2.03 (t, 1H, *J* = 2.7, CC*H*); ^{13}C NMR (150 MHz, $CDCl_3$) δ_C 195.7 (*C*(O)), 134.6 (Ar*C*H), 129.7 (Ar*C*H), 129.4 (Ar*C*H), 127.4 (Ar*C*), 82.0 (*C*), 69.7 (*C*H), 42.1 (C(O)*C*H_2), 14.7 (C(O)CH_2*C*H_2); IR (oil) v_{max}/ cm^{-1} 3292 (m), 2956 (w), 2921 (w), 1702 (s), 1582 (w); LRMS (ES+) *m/z* 191 ([M+H]$^+$, 100), 175 (9); HRMS (ES+) calcd for $[C_{11}H_{11}OS]^+$ [M+H]$^+$ 191.0525, observed 191.0527.

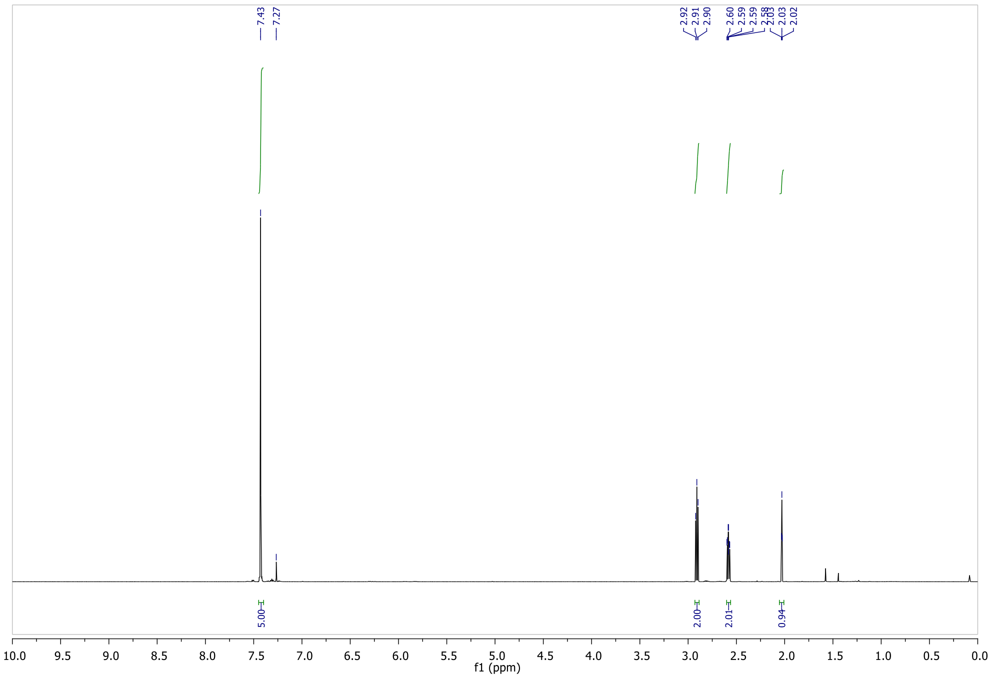

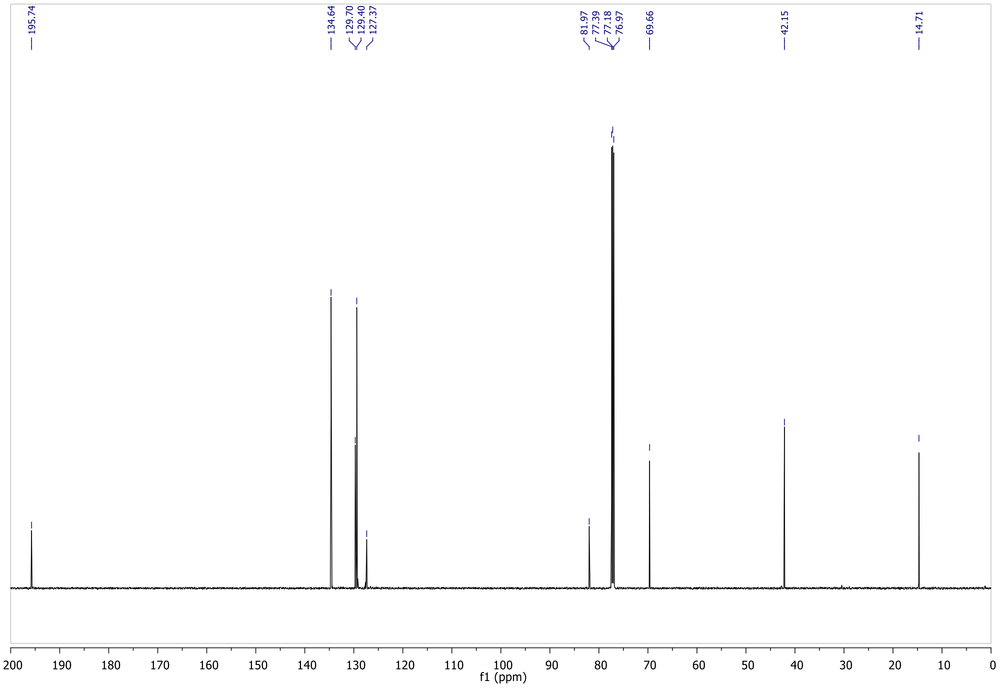

Sodium 2-(pent-4-ynoylthio)ethane-1-sulfonate (3)

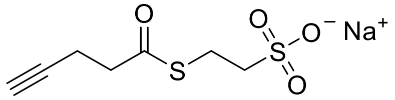

To 4-pentynoic acid (200 mg, 2.04 mmol) in MeCN (15 mL) and DMF (2 mL) was added EEDQ (604 mg, 2.45 mmol) and the resultant mixture was stirred at RT for 30 min. Then, sodium 2-mercaptoethanesulfonate (335 mg, 2.04 mmol) was added and the resultant mixture was stirred at 80 ^{o}C for 18 h. The solvent was then removed *in vacuo* and purification by column chromatography (gradient elution from CH_2Cl_2 to 20% MeOH in CH_2Cl_2) afforded the target compound as a white solid (357 mg, 1.46 mmol, 72%).

mp 240 ^{o}C (decomposition); ^{1}H NMR (600 MHz, CD_3OD) δ_H 3.27-3.24 (m, 2H, C(O)SC*H*$_2$), 3.01-2.98 (m, 2H, C*H*$_2$$SO_3$Na), 2.78 (t, 2H, *J* = 7.2, C(O)C*H*$_2$), 2.50 (td, 2H, *J* = 7.2, 2.5, C(O)CH_2C*H*$_2$), 2.27 (t, 1H, *J* = 2.3, C*H*); ^{13}C NMR (150 MHz, CD_3OD) δ_C 198.4 (*C*(O)), 82.8 (*C*), 70.5 (*C*H), 52.2 (*C*H_2SO_3Na), 43.2 (C(O)*C*H_2), 25.0 (C(O)S*C*H_2), 15.2 (C(O)CH_2*C*H_2); IR (solid) v_{max}/ cm^{-1} 3291 (m), 3237 (m), 2993 (w), 2938 (w), 1679 (s); LRMS (ES-) *m/z* 221 ([M-H]$^-$, 100); HRMS (ES-) calcd for $[C_7H_8O_4S_2]^-$ $[M-H]^-$ 220.9948, observed 220.9945.

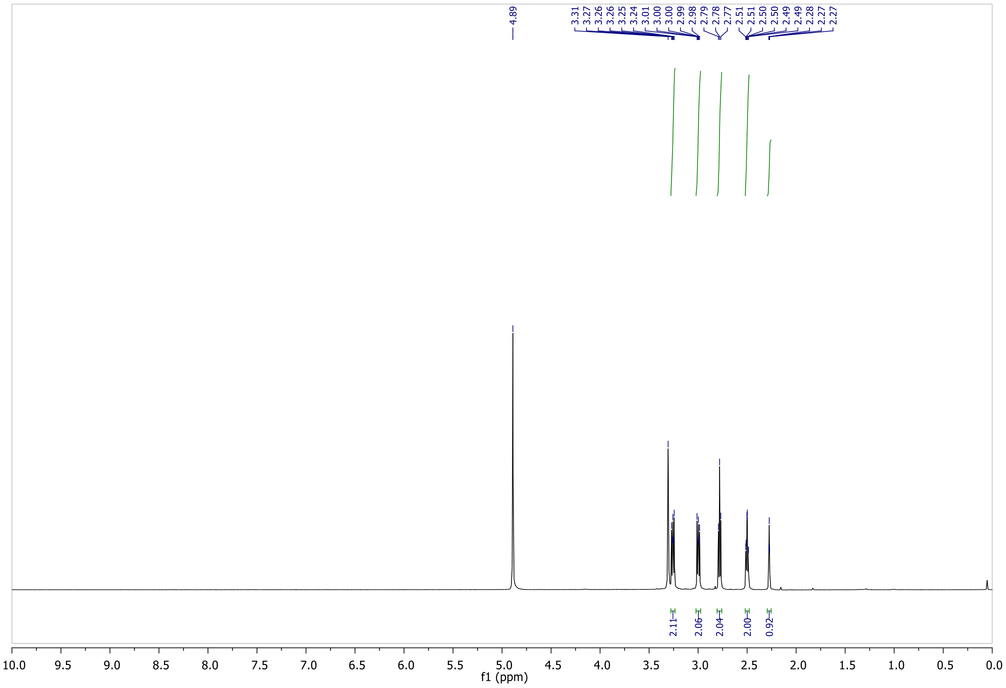
4.89
3.31
3.27
3.26
3.26
3.25
3.24
3.01
3.00
3.00
2.99
2.98
2.79
2.78
2.77
2.51
2.51
2.50
2.50
2.49
2.49
2.28
2.27
2.27
2.11
2.06
2.04
2.00
0.92
f1 (ppm)

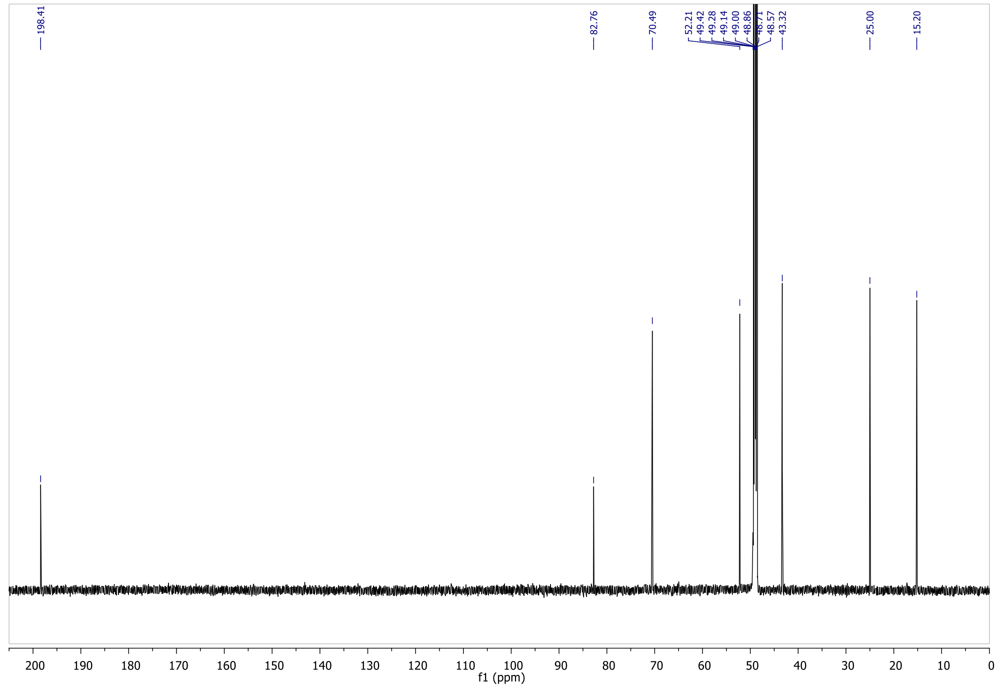
198.41
82.76
70.49
52.21
49.42
49.28
49.14
49.00
48.86
48.71
48.57
43.32
25.00
15.20
f1 (ppm)

2-(prop-2-yn-1-yl)malonic acid (11)

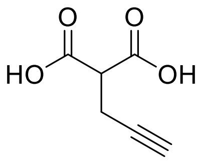

Dimethyl propargylmalonate (894 µL, 5.88 mmol) was dissolved in MeOH (25 mL). NaOH (24 mL, 1M aq. solution) was then added and the resultant orange solution was stirred at RT for 90 min. After this period, the mixture was acidified to pH 2 with HCl (1M aq. solution) and extracted into EtOAc (3 × 50 mL). The organic layer was dried (Na_2SO_4) and concentrated *in vacuo* to give the target compound as an off white solid (715 mg, 5.03 mmol, 86%).

mp 133-135 ºC, ^{1}H NMR (600 MHz, CD_3OD) δ_H 3.50 (t, 1H, *J* = 7.6, C(O)C*H*C(O)), 2.69 (dd, 2H, *J* = 7.6, 1.5, CHCH_2), 2.31 (s, 1H, CC*H*); ^{13}C NMR (150 MHz, CD_3OD) δ_C 171.3 (*C*(O), 81.3 (*C*CH), 71.1 (C*C*H), 52.4 (C(O)*C*HC(O)), 19.1 (CH*C*H_2); IR (solid) ν_{max}/ cm^{-1} 3304 (w), 2851 (w, br), 1696 (s); LRMS (ES+) *m/z* 165 ([M+Na]$^+$, 100), 143 ([M+H]$^+$, 14), 125 (52); HRMS (ES+) calcd for $[C_6H_7O_4]^+$ [M+H]$^+$ 143.0339, observed 143.0342.

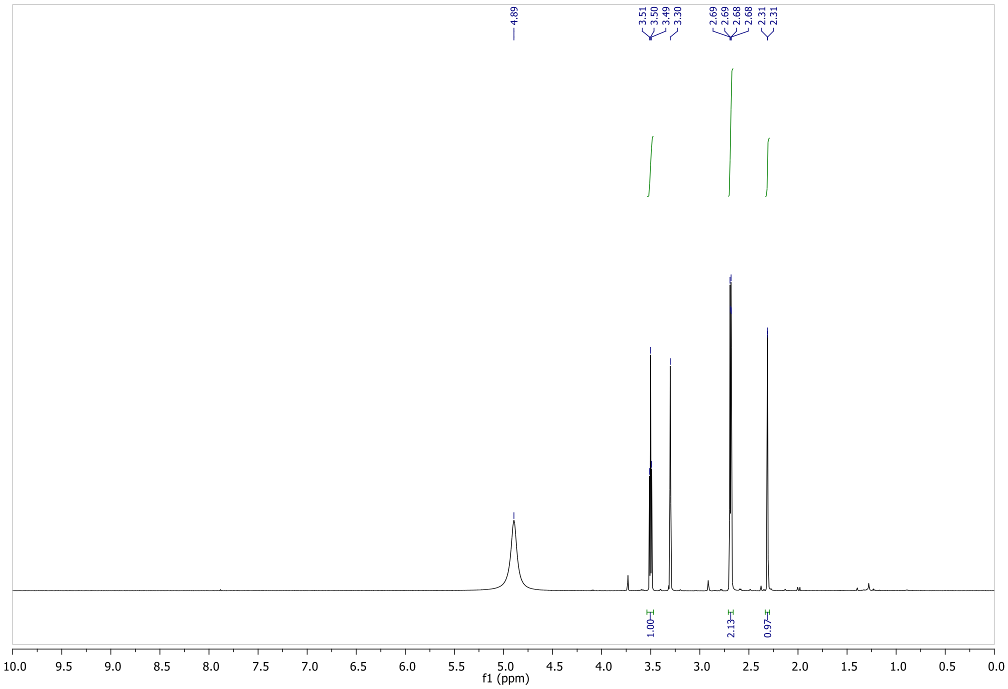

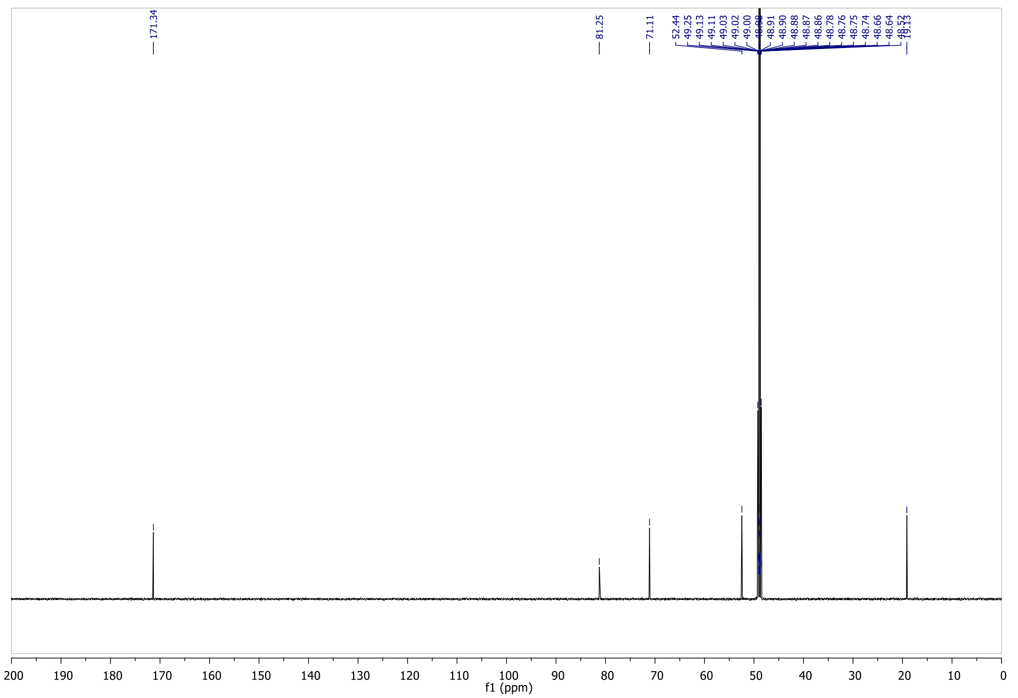

S,S-bis(2-methoxyethyl) 2-(prop-2-yn-1-yl)propanebis(thioate) (7)

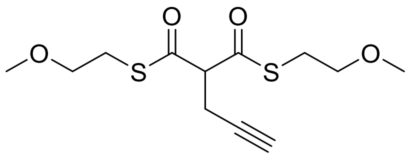

To 2-(prop-2-yn-1-yl)malonic acid **11** (100 mg, 0.704 mmol) in CH_2Cl_2 (7 mL) was added oxalyl chloride (775 μL, 1.55 mmol, 2 M solution in CH_2Cl_2), followed by a catalytic amount of DMF (2 drops). After 9 h at RT, 2-methoxyethanethiol (166 μL, 1.69 mmol) was added, followed by NEt_3 (230 μL, 1.69 mmol) and the resultant mixture was stirred at RT for 12 h. The solvent was then removed *in vacuo* and purification by column chromatography (gradient elution pet. ether to 60% EtOAc in pet. ether) afforded the target compound as a colourless oil (126 mg, 0.434 mmol, 62%).

^{1}H NMR (600 MHz, $CDCl_3$) δ_H 3.99 (t, 1H, *J* = 7.5, C(O)C*H*C(O)), 3.51 (t, 4H, *J* = 6.2, 2 × C*H*$_2$OCH_3), 3.34 (s, 6H, 2 × OC*H*$_3$), 3.18-3.10 (m, 4 H, 2 × SC*H*$_2$), 2.80 (dd, 2H, *J* = 7.5, 2.7, CHC*H*$_2$), 2.02 (s, 1H, CC*H*); ^{13}C NMR (150 MHz, $CDCl_3$) δ_C 192.2 (S*C*(O)), 79.3 (*C*CH), 71.2 (C*C*H), 70.7 (*C*H$_2$OCH_3), 66.3 (C(O)*C*HC(O)), 58.9 (O*C*H$_3$), 29.6 (S*C*H$_2$), 19.3 (CH*C*H$_2$); IR (oil) ν_{max}/ cm^{-1} 3280 (w), 2927 (w), 2887 (w), 2824 (w), 1693 (s), 1666 (s); LRMS (ES+) *m/z* 308

([M+NH_4]$^+$, 15), 291 ([M+H]$^+$, 100), 214 (25), 193 (21), 165 (14); HRMS (ES+) calcd for [$C_{12}H_{19}O_4S_2$]$^+$ [M+H]$^+$ 291.0729, observed 291.0725.

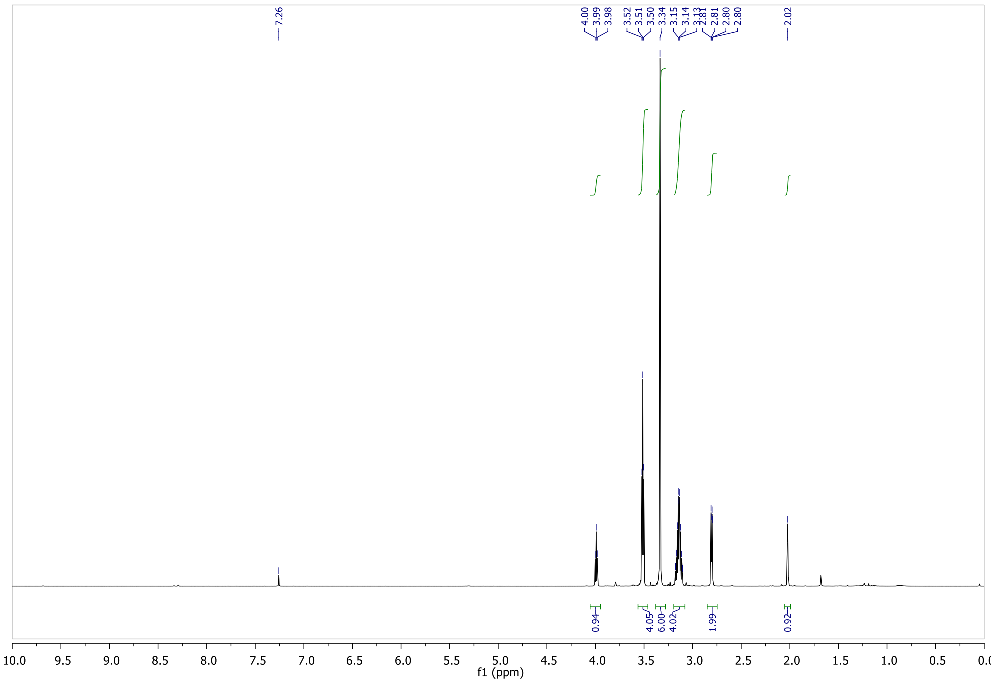

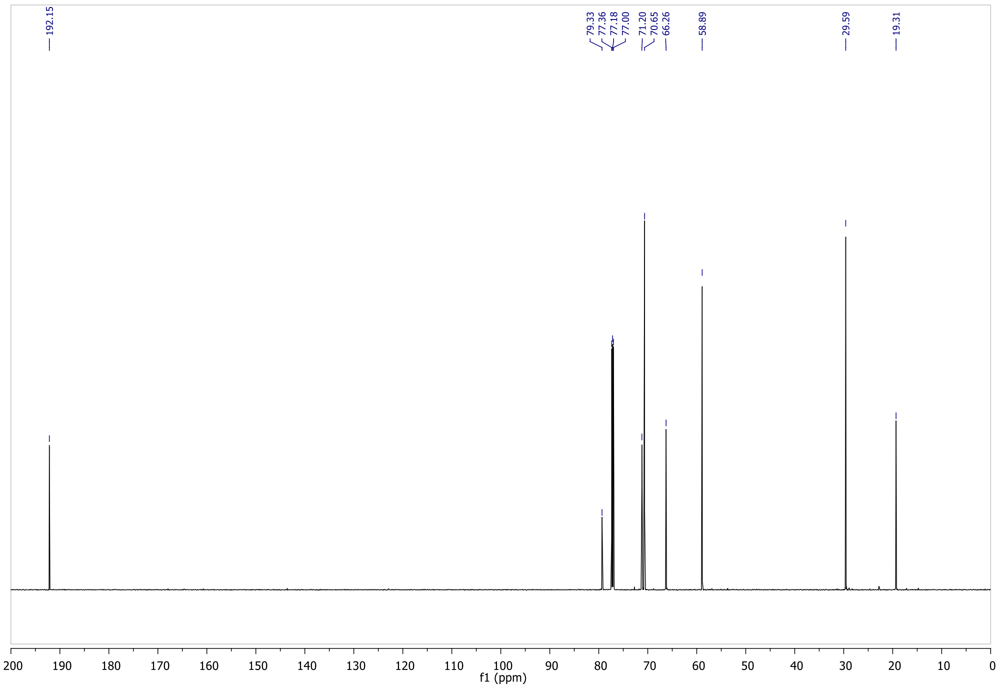

Bioconjugation general remarks

Conjugation experiments were carried out in standard polypropylene Eppendorf safe-lock tubes (1.5 or 2.0 mL) at atmospheric pressure with mixing at the temperature stated. Reagents and solvents were purchased from commercial sources and used as supplied. All buffer solutions were prepared with doubly deionized water and filter-sterilized. All buffer solutions were degassed prior to use, where the term 'degassed' refers to the process of removing O_2 from a solution by bubbling argon through it. Conjugation buffer was 40 mM phosphates, 20 mM NaCl, 6 mM EDTA at pH 7.4. Borate-buffered saline (BBS) was 80 mM boric acid, 20 mM NaCl at the specific pH. Phosphate-buffered saline (PBS) was 12 mM phosphates, 140 mM NaCl at pH 7.4. Ultrapure DMF was purchased from Sigma and stored under dry conditions. Ultrafiltration was carried out in Amicon® Ultra-4 Centrifugal Filter Units with a molecular weight cut-off (MWCO) of 10 kDa or in Vivaspin® 500 centrifugal concentrators (10 kDa MWCO). Centrifugation was carried out on an eppendorf 5415R fixed angle rotor centrifuge operating at 14000 rcf at 20 °C or in an eppendorf 5810 swing-bucket rotor centrifuge operating at 3220 rcf at 20 °C. Concentration of peptide samples following digestion was carried out using an Eppendorf® centrifugal vacuum concentrator 5301 operating at 240 rcf at 45 °C. Size exclusion chromatography (SEC) was carried out on an ÄKTA FPLC system (GE Healthcare), equilibrated in PBS. Detection was by absorption at 280 nm. Trastuzumab (Herceptin™) was purchased from UCLH in its clinical formulation (Roche, lyophilised). Herceptin Fab was prepared by a sequential enzymatic digest of the full antibody with pepsin and papain, following a literature procedure.[2] Concentrations were determined by UV/ Vis absorbance using molecular extinction coefficient of ε_{280} = 68590 M^{-1} cm^{-1} for trastuzumab Fab.[3] Treatment of CLT conjugates with BME was shown to result in a small amount of disulfide bond reduction; reoxidation was shown to be effected by overnight incubation of the sample at 4 °C prior to LC-MS analysis, or by addition of DTNB (10 eq., 5 min, 20 °C).[4] Protein conjugation reactions were monitored by 12% glycine-SDS-PAGE with a 6% stacking gel under non-reducing conditions, unless otherwise stated. Samples were mixed 1:1 with SDS non-reducing loading buffer (composition for 6 x SDS: 1 g SDS, 3 mL glycerol, 6 mL 0.5 M Tris buffer pH 6.8, 2 mg bromophenol blue) and heated at 75 °C for 5 min before applied to the gel. Samples were run at constant current (30 mA) for 40 minutes in 1 x SDS running buffer. Gels were stained with Coomassie G-250 (0.05% w/v) in 49.95% H_2O, 40% MeOH, 10% AcOH and de-stained with

10% MeOH, 10% AcOH, 80% H_2O solution. Absorbance measurements were carried out on a Carry Bio 100 UV/ Vis spectrophotometer (Varian) equipped with a temperature-controlled 12x sample holder in quartz cuvettes (Starna Scientific - 1 cm path length, volume 160 µL) at RT. Samples were baseline corrected. The UV data was analyzed using Graphpad Prism 7.03 software.

LC-MS general remarks

Determination of Protein Masses by LC-MS

Molecular masses of native and modified proteins were measured using an Agilent 6510 QTOF LC-MS system (Agilent, UK). Agilent 1200 HPLC system was equipped with an Agilent PLRP-S, 1000A, 8 µM, 150 mm x 2.1 mm column. 10 µL of a protein sample (at *ca.* 2-4 µM) was separated on the column using mobile phase A (water-0.1% formic acid) and B (acetonitrile-0.1% formic acid) with an eluting gradient (as shown in Table S1) at a flow rate of 300 µl/min. The oven temperature was maintained at 60 °C.

Time (min)	Solvent A (%)	Solvent B (%)
0	85	15
2	85	15
3	68	32
4	68	32
14	65	35
18	5	95
20	5	95
22	85	15
25	85	15

Table S1 - LC-MS mobile phase A/ B gradient elution.

Agilent 6510 QTOF mass spectrometer was operated in a positive polarity mode, coupled with an ESI ion source. The ion source parameters were set up with a VCap of 3500V, a gas temperature at 350 °C, a dry gas flow rate at 10 L/min and a nebulizer of 30 psig. MS Tof was acquired under conditions of a fragmentor at 350 V, a skimmer at 65 V and an acquisition rate at 0.5 spectra/s in a profile mode, within a scan range between 700 and 5000 m/z. The .d data was then analysed by

deconvoluting a spectrum to a zero charge mass spectra using a maximum entropy deconvolution algorithm within the MassHunter software version B.07.00.

Capillary LC-MS/MS analysis

The LC-MS/MS system consisted of an 1120 series liquid chromatograph system coupled to a 6510 QTOF mass spectrometer with a dual ESI probe (all Agilent Technologies, UK). Chromatographic separation of peptides was achieved on a ZORBAX Extend 300 C18, 2.1 x 100 mm, analytical column packed with 3.5 µm particles. Peptides were loaded onto a ZORBAX Extend 300 C18, 2.1 x 12.5 mm, 5 µm particles guard cartridge with 99% solvent A (H_2O/ 0.1% formic acid) and 1% B (MeCN/ 0.1% formic acid). They were then eluted with a gradient from 1% B to 40% B in 35 min, followed by a steep gradient to 95% B in 0.1 min and stayed at 95% B for 4.9 min. At 40.1 min B was returned to 1% for 4.9 min to recondition the LC column for the next injection. The flow rate was 270 µL min^{-1}. The total run time was 50 min.

The LC eluent was continuously directed to the dual ESI source of the QTOF mass spectrometer operating in positive mode. Data were acquired using data-dependent MS/MS acquisition. The ESI source parameters for MS and MS/MS acquisitions were: gas temperature 350°C, nitrogen gas flow 10 L/min, nebuliser 35 psi, VCap 4000, fragmentor 175, skimmer 65 and octopole RF peak 750, ion control was on. MS scan range was 300 to 2500 Da with 4.1 scan rate. MS/MS range was 50-3000 Da with scan rate 3. MS scans were acquired at a resolution of 25,000 at 922 Da with 5 maximum precursors per cycle with threshold abundance set at 1000 for peptide ions with multiply charge state ($z = 2, 3, \geq 3$) which were selected with narrow isolation width at ~1.3 Da for MS/MS fragmentation. The ramped collision energy was used with slope 3.6 and offset -4.8. MS/MS scans were acquired at a resolution of 17,500 at 922 Da. Data was stored in centroid mode.

Database search

The Herceptin amino acids sequence was obtained from the SwissProt database 2018. The in-house database was created for light and heavy chains of Herceptin in the Agilent Spectrum Mill Server software (version 2.2.3) installed on a dual Xeon 2.4-GHz computer. Peak lists were created with the Spectrum Mill Data Extractor program with the following attributed:- scans with the same precursor ± 1.4 *m/z* were merged within a time frame of ± 15 s. Precursor ions needed to have a minimum signal to noise value of 25. Charges up to a maximum of 7 were assigned to the precursor ion, and the ^{12}C peak was determined by the Data Extractor. The Herceptin in-house database was searched for peptides with a mass tolerance of 20 ppm for the precursor ions and a tolerance of 30 ppm for the fragment ions. Four missed cleavages were allowed. The conjugated lysine peptides were identified using fixed modification of carbamidomethylation of cysteine residues, variable deamidation modification of asparagine and glutamine, methionine oxidation and conjugation of lysine with either C_5H_4O (non-bridged system) or $C_6H_4O_3$ (bridged system). The dynamic peak thresholding search mode was variable modifications with precursor mass shift range from 0 to 500 Da. A Spectrum Mill auto validation was performed first in the protein details mode. Minimum scores, minimum scored peak intensity (SPI), forward minus reversed score thresholds. Then auto validation in the peptide mode was performed using a score threshold of 10 and SPI of 60% for 1+, 2+, 3+ and 4+ and 5+. Forward minus reversed score threshold and rank 1 minus rank 2 score threshold were set to 2. MS/MS spectra of all measured lysine modified peptides were checked also manually for a-, b- and y-ions.

LC-MS analysis of native Fab and reduced Fab

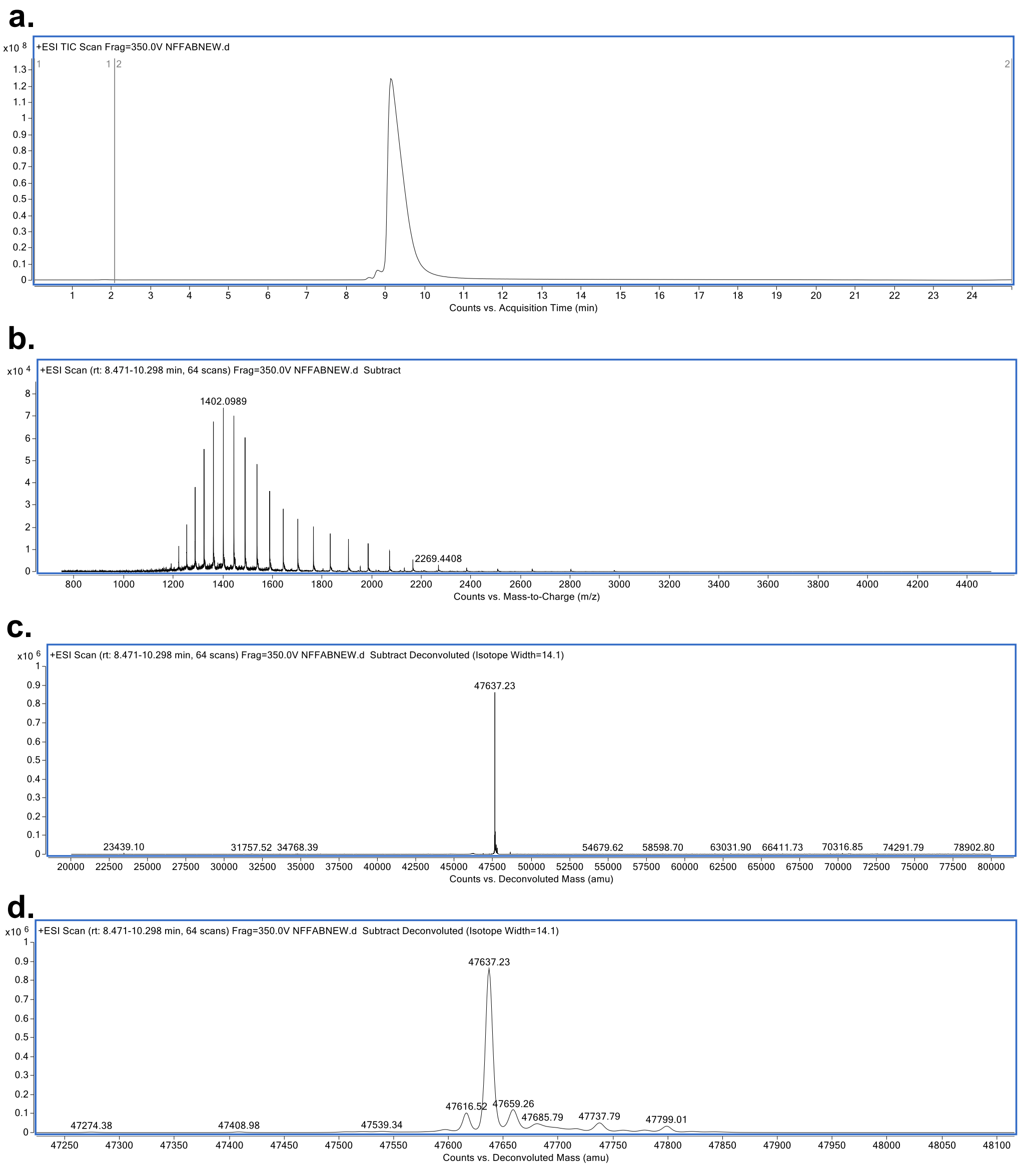

Figure S1 - LC-MS analysis of native anti-HER2 Fab; a. TIC, b. non-deconvoluted ion-series c. deconvoluted ion series mass spectrum; observed mass of 47637, d. zoomed in deconvoluted ion series mass spectrum.

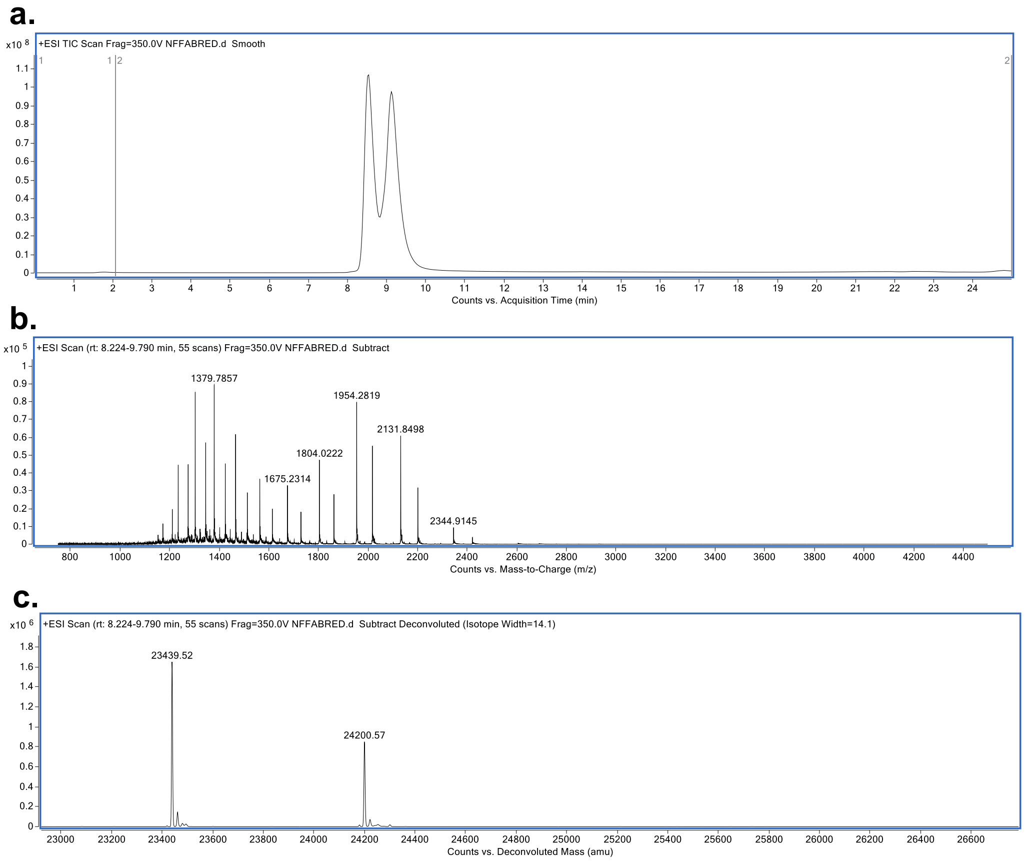

Figure S2 - LC-MS analysis of reduced native anti-HER2 Fab (reduction with 10 eq. TCEP, 1 h, 37 ºC); a. TIC, b. non-deconvoluted ion-series c. deconvoluted ion series mass spectrum; observed mass of light chain 23440, observed mass of heavy chain 24201.

Reaction of anti-HER2 Fab with NHS-ester alkyne 1

To anti-HER2 Fab (30 μL, 0.0018 μmol, 60 μM, 2.85 mg/mL) in conjugation buffer was added NHS-ester alkyne **1** (0.30 μL, 0.0045 μmol, 15 mM solution in DMF, 2.5 eq.). The resultant mixture was incubated at 4 °C for 16 h. After this period, the excess reagent was removed *via* ultrafiltration (10 kDa MWCO) into H_2O and the sample submitted for LC-MS analysis.

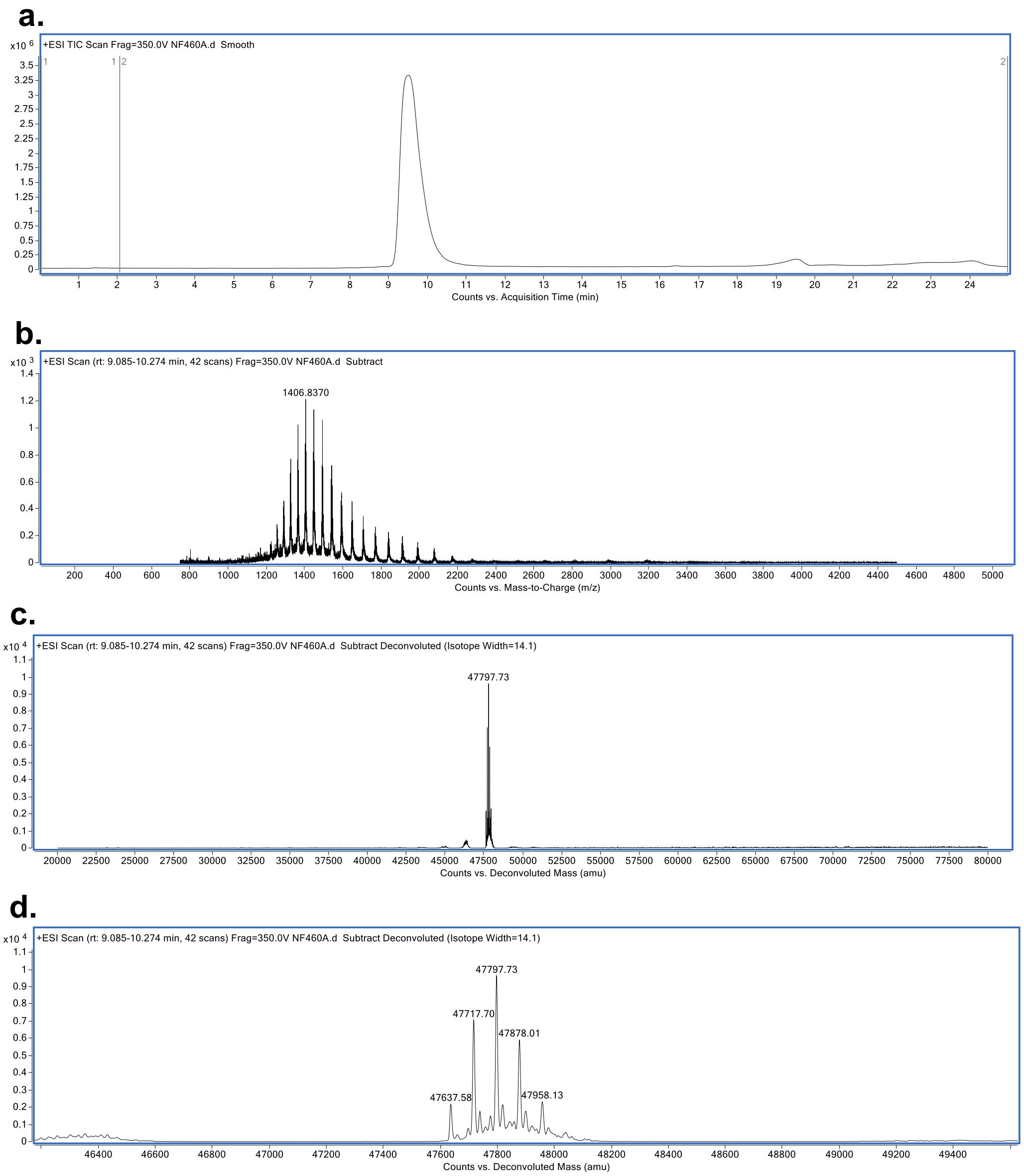

Figure S3 - LC-MS analysis of anti-HER2 Fab reacted with NHS-ester alkyne **1**; a. TIC, b. non-deconvoluted ion-series c. deconvoluted ion series mass spectrum, d. zoomed in deconvoluted ion series

mass spectrum; observed mass of 47638 corresponds to native Fab, 47718 corresponds to one acyl addition (expected 47718), 47798 corresponds to two acyl additions (expected 47798), 47878 corresponds to three acyl additions (expected 47878), 47958 corresponds to four acyl additions (expected 47958).

Reaction of reduced anti-HER2 Fab with NHS-ester alkyne 1

To anti-HER2 Fab (50 μL, 0.0030 μmol, 60 μM, 2.85 mg/mL) in conjugation buffer was added TCEP (0.50 μL, 0.030 μmol, 60 mM solution in H_2O, 10 eq.). After 1 h at 37 ^oC, NHS-ester alkyne **1** (0.25 μL, 0.0075 μmol, 30 mM solution in DMF, 2.5 eq.) was added in the conjugation and the resultant mixture was incubated at 22 ^oC for 1 h. The excess reagent was then removed *via* ultrafiltration (10 kDa MWCO) into H_2O and the sample submitted for LC-MS analysis.

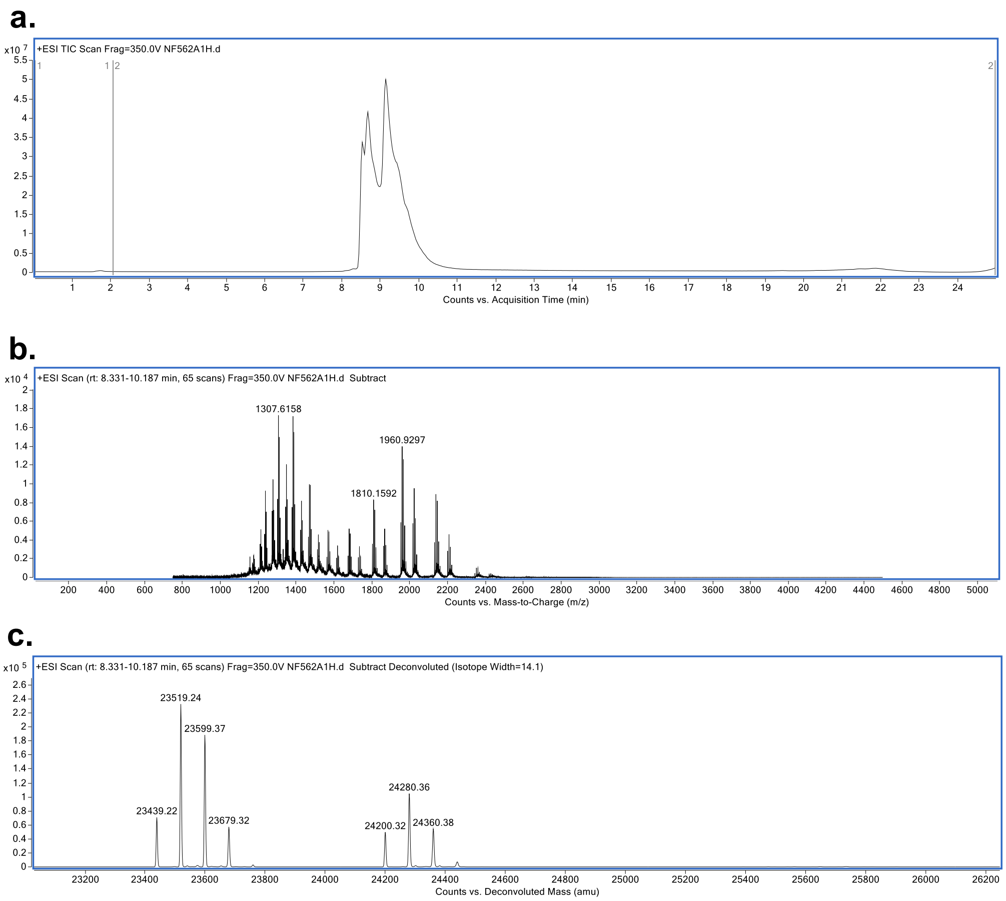

Figure S4 - LC-MS analysis of reduced anti-HER2 Fab reacted with NHS-ester alkyne **1**; a. TIC, b. non-deconvoluted ion-series c. deconvoluted ion series mass spectrum; observed mass of 23439 corresponds

to LC, 23519 corresponds to one acyl addition on LC (expected 23520), 23599 corresponds to two acyl additions on LC (expected 23600), 23679 corresponds to three acyl additions on LC (expected 23680), 24200 corresponds to HC, 24280 corresponds one acyl addition on HC (expected 24281), 24360 corresponds to two acyl additions on HC (expected 24361).

Reaction of anti-HER2 Fab with aryl thioester 2

Herceptin Fab (100 µL, 0.00372 µmol, 37.2 µM, 1.77 mg/mL) in conjugation buffer was incubated with aryl thioester **2** (1.86 µL, 0.0370 µmol, 20 mM solution in DMF, 10 eq.) at RT for 15 min. The excess thioester was then removed *via* ultrafiltration (10 kDa MWCO) into H_2O and the sample submitted for LC-MS analysis.

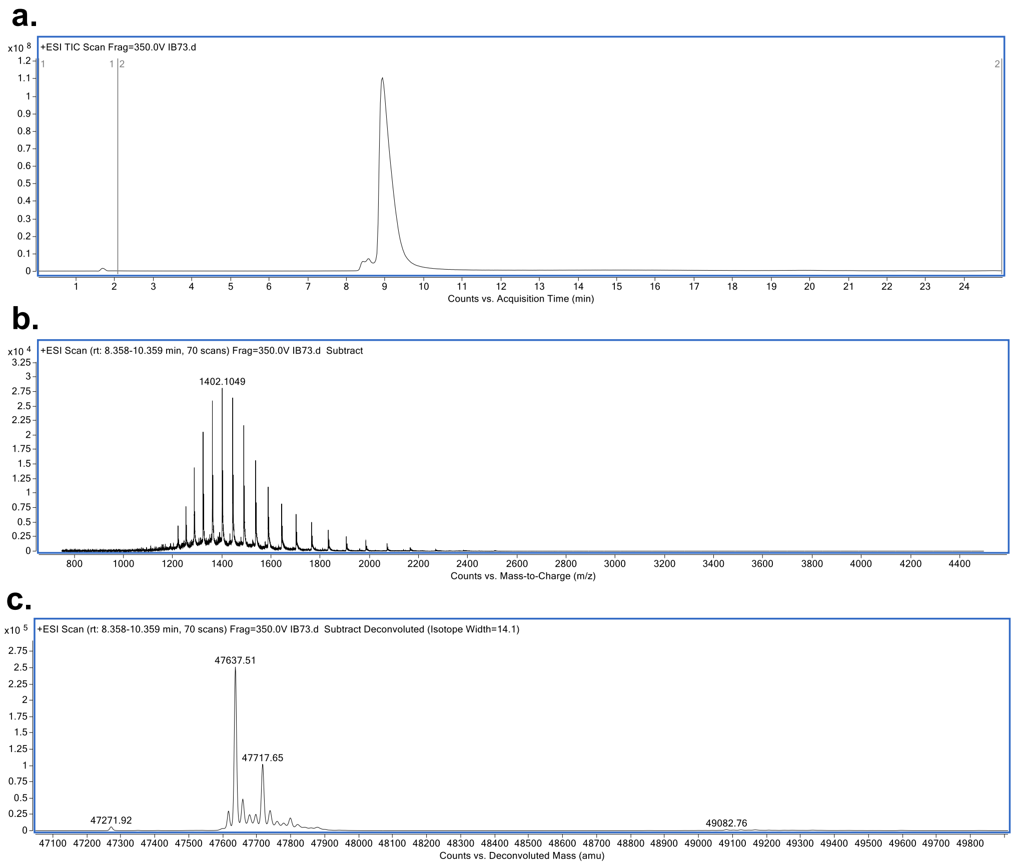

Figure S5 - LC-MS analysis of control reaction of anti-HER2 Fab with aryl thioester **2** (10 eq., 15 min, RT, pH 7.4); a. TIC, b. non-deconvoluted ion-series c. deconvoluted ion series mass spectrum; observed mass of 47638 corresponds to native Fab, 47718 corresponds to one acyl addition (expected 47718).

Reaction of anti-HER2 Fab with aryl thioester 2, followed by reduction with TCEP

Herceptin Fab (150 µL, 0.00558 µmol, 37.2 µM, 1.77 mg/mL) in conjugation buffer was incubated with aryl thioester **2** (2.79 µL, 0.0558 µmol, 20 mM solution in DMF, 10 eq.) at RT for 15 min. The excess thioester was then removed *via* ultrafiltration (10 kDa MWCO) into conjugation buffer. The concentration was determined by UV/Vis absorbance and adjusted to 37.2 µM (120 µL, 1.77 mg/mL). To this solution was added TCEP (2.23 µL, 0.0446 µmol, 20 mM solution in H_2O, 10 eq.). After 1 h at 37 °C, the excess reagent was removed *via* ultrafiltration into H_2O (10 kDa MWCO) and the sample submitted for LC-MS analysis.

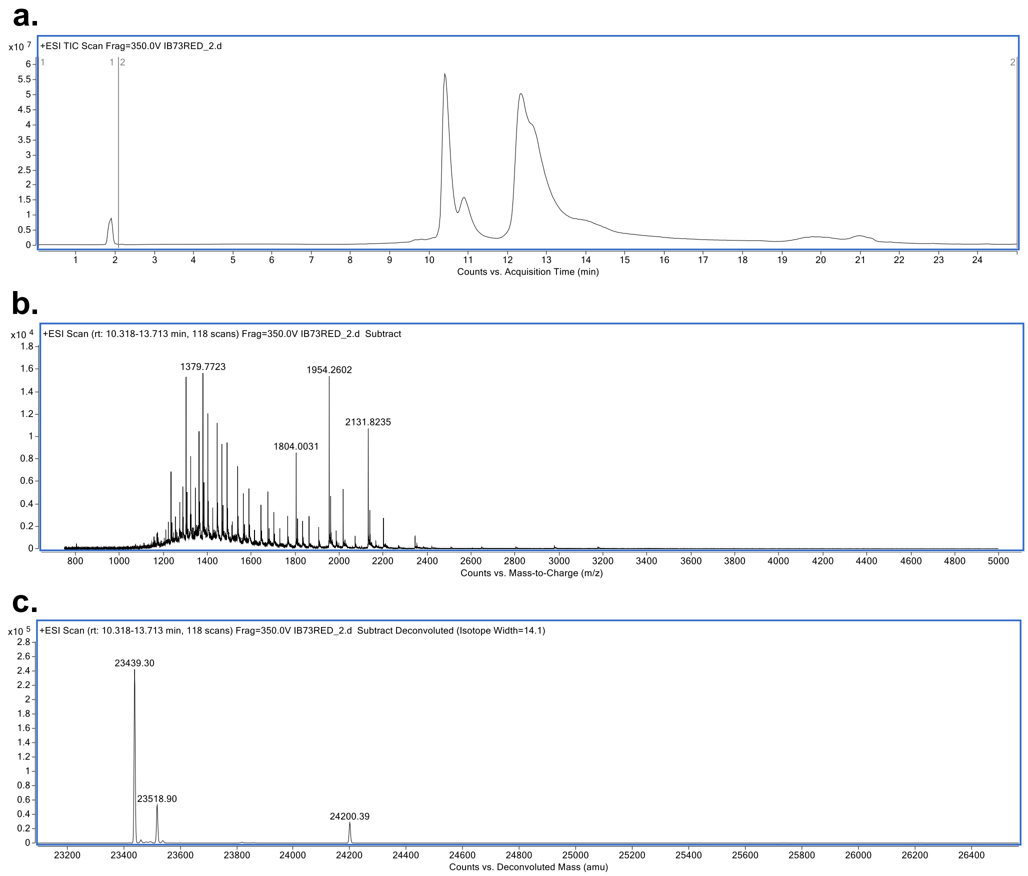

Figure S6 - LC-MS analysis of control reaction of anti-HER2 Fab with aryl thioester **2**, followed by TCEP reduction; a. TIC, b. non-deconvoluted ion-series c. deconvoluted ion series mass; observed mass of 23439 corresponds to native LC, 23519 corresponds to one acyl addition on LC (expected 23520), 24200 corresponds to native HC.

Reaction of reduced anti-HER2 Fab with aryl thioester 2

Herceptin Fab (100 μL, 0.00372 μmol, 37.2 μM, 1.77 mg/mL) in conjugation buffer was incubated with TCEP (3.72 μL, 0.0372 μmol, 10 mM solution in H_2O, 10 eq.). After 1 h at 37 °C, aryl thioester **2** (1.86 μL, 0.0370 μmol, 20 mM solution in DMF, 10 eq.) was added in the conjugation and the resultant mixture was incubated at RT for 15 min. The excess thioester was then removed *via* ultrafiltration (10 kDa MWCO) into H_2O and the sample submitted for LC-MS analysis.

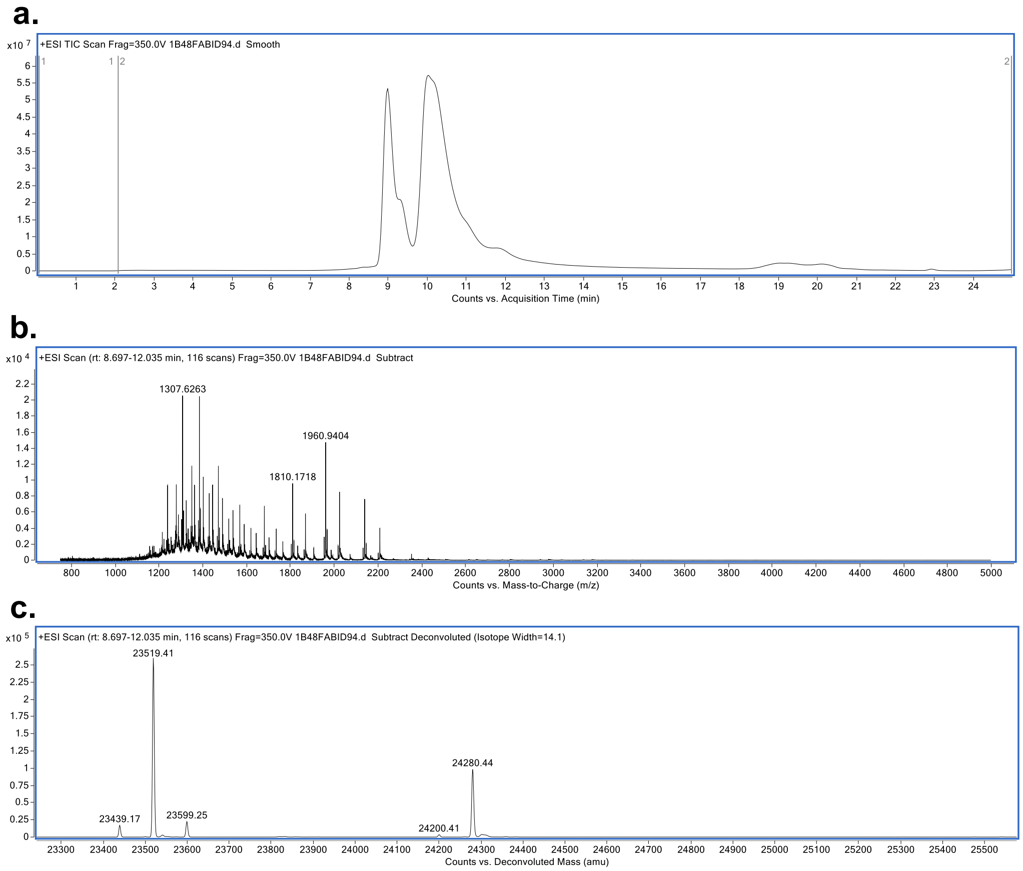

Figure S7 - LC-MS analysis of reduced Fab reacted with aryl thioester **2** (10 eq., 15 min, pH 7.4, RT); a. TIC, b. non-deconvoluted ion-series c. deconvoluted ion series mass spectrum; observed mass of 23439 corresponds to LC, 23519 corresponds to one acyl addition on LC (expected 23520), 23599 corresponds to two acyl additions on LC (expected 23600), 24280 corresponds to one acyl addition on HC (expected 24281).

Reaction of anti-HER2 Fab with MESNa thioester 3

To anti-HER2 Fab (40 µL, 0.0060 µmol, 150 µM, 7.15 mg/mL) in conjugation buffer was added thioester **3** (2.0 µL, 0.60 µmol, 300 mM solution in DMF, 100 eq.) and the resultant mixture was incubated at 22 °C for 4 h. The excess thioester was then removed *via* ultrafiltration (10 kDa MWCO) into H_2O and the sample submitted for LC-MS analysis.

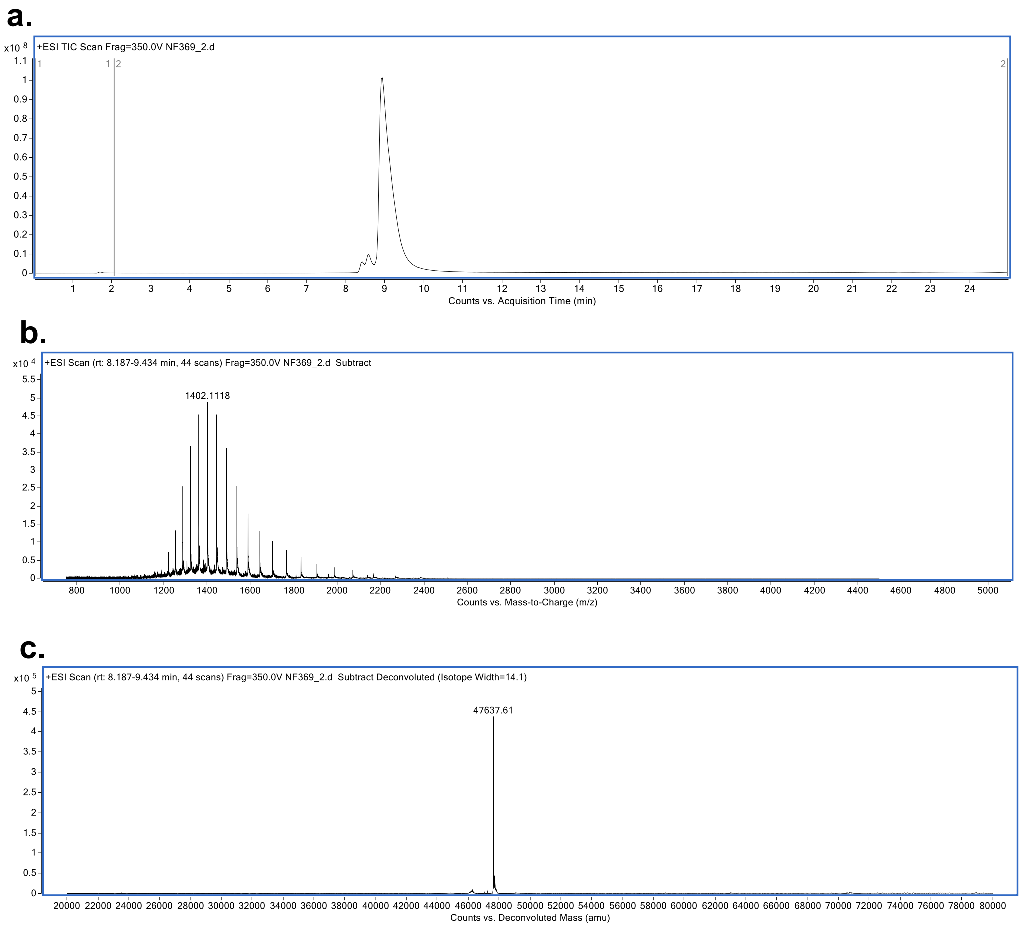

d.

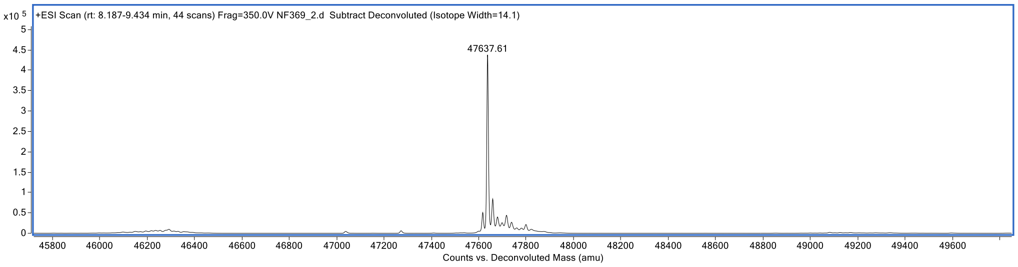

Figure S8 - LC-MS analysis of control reaction of anti-HER2 Fab with MESNa thioester **3** (100 eq., 4 h, 22 ^{o}C); a. TIC, b. non-deconvoluted ion-series c. deconvoluted ion series mass spectrum, d. zoomed in deconvoluted ion series mass spectrum; observed mass of 47638 corresponds to native Fab.

Transthioesterification of anti-HER2 Fab with MESNa thioester 3 (thioester conjugate 4)

To anti-HER2 Fab (40 µL, 0.0060 µmol, 150 µM, 7.15 mg/mL) in conjugation buffer was added TCEP (0.4 µL, 0.060 µmol, 150 mM solution in H_2O, 10 eq.). After 1 h at 37 ^{o}C, thioester **3** (2.0 µL, 0.60 µmol, 300 mM solution in DMF, 100 eq.) was added to the conjugation and the resultant mixture was incubated at 22 ^{o}C for 4 h. The excess thioester was then removed *via* ultrafiltration (10 kDa MWCO) into H_2O and the sample submitted for LC-MS analysis, where it was kept at 4 ^{o}C until analyzed.

a.

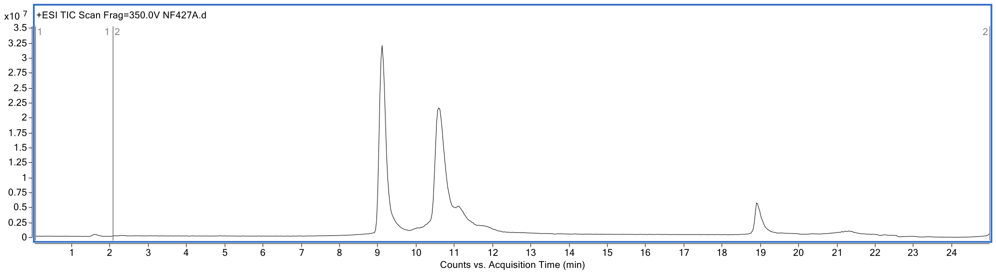

b.

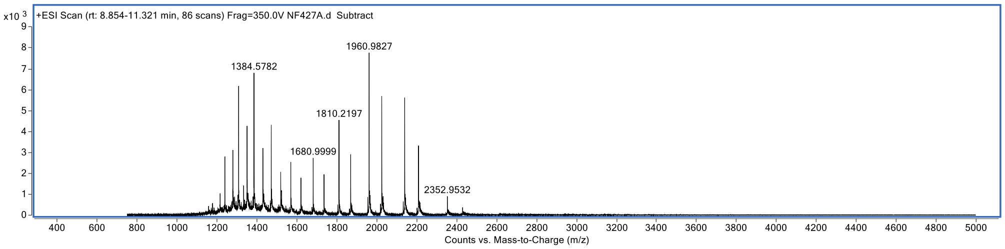

c.

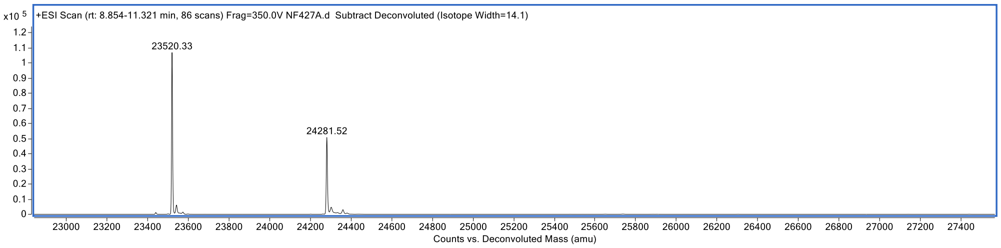

Figure S9 - LC-MS analysis of thioester conjugate **4** (transthioesterification of anti-HER2 Fab with MESNa thioester **3**); a. TIC, b. non-deconvoluted ion-series c. deconvoluted ion series mass spectrum; observed mass of 23520 corresponds to one acyl addition on LC (expected 23520), 24282 corresponds to one acyl addition on HC (expected 24281).

Treatment of thioester conjugate 4 with cysteine

To anti-HER2 Fab after transthioesterification with MESNa thioester **3** (280 µL, 0.00560 µmol, 0.95 mg/mL, 20 µM) in conjugation buffer was added L-Cysteine.HCl (9.33 µL, 0.560 µmol, 60 mM solution in conjugation buffer, 100 eq.). After 2 h at 22 ^{o}C, the excess L-Cysteine.HCl was removed *via* ultrafiltration (10 kDa MWCO) into H_2O and the sample submitted for LC-MS analysis.

a.

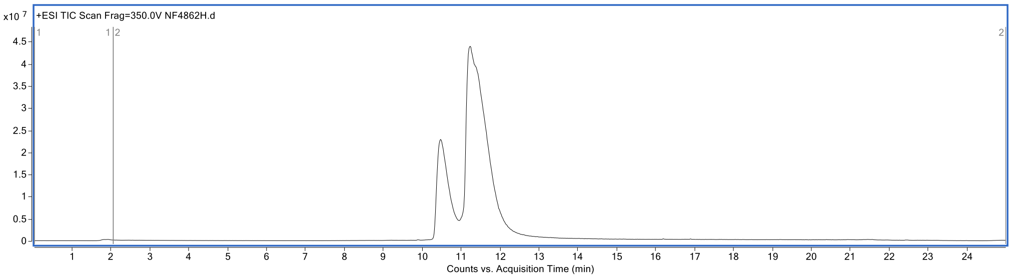

b.

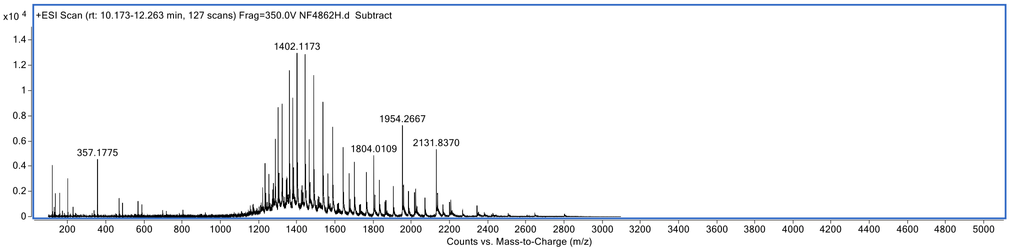

c.

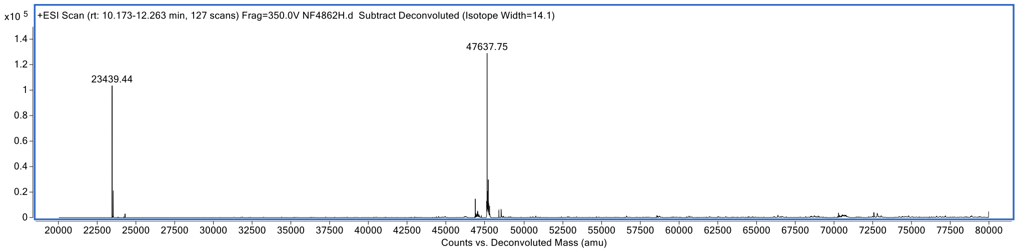

Figure S10 - LC-MS analysis of thioester conjugate **4** treated with cysteine; a. TIC, b. non-deconvoluted ion-series c. deconvoluted ion series mass spectrum; observed mass of 23439 corresponds to native LC, 47638 corresponds to native Fab.

Optimized conditions for acyl transfer of thioester conjugate 4 at pH 8.4, 12 ^{o}C, 72 h (CLT conjugate 5)

To anti-HER2 Fab (330 μL, 0.0495 μmol, 150 μM, 7.15 mg/mL) in conjugation buffer was added TCEP (3.30 μL, 0.495 μmol, 150 mM solution in H_2O, 10 eq.). After 1 h at 37 ^{o}C, thioester **3** (16.5 μL, 4.95 μmol, 300 mM solution in DMF, 100 eq.) was added in the conjugation and the resultant mixture was incubated at 22 ^{o}C for 4 h. The excess thioester was then removed *via* ultrafiltration (10 kDa MWCO) into conjugation buffer and buffer exchanged into BBS buffer (pH 8.4). The concentration was determined by UV/Vis absorbance and adjusted to 20 μM (2068 μL, 0.95 mg/mL). The resultant solution was incubated for 72 h at 12 ^{o}C. After this period, the sample was buffer exchanged into H_2O *via* ultrafiltration (10 kDa MWCO) and submitted for LC-MS analysis.

a.

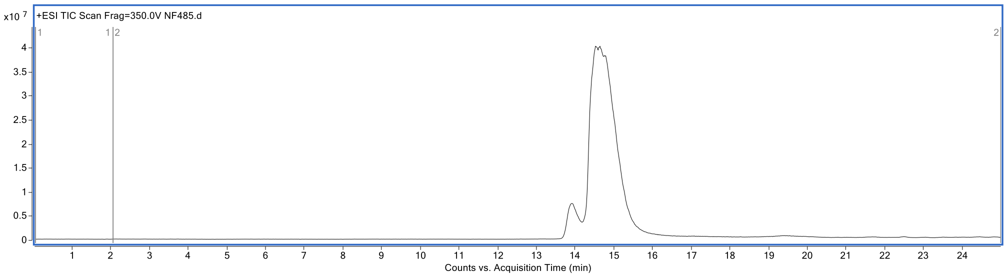

b.

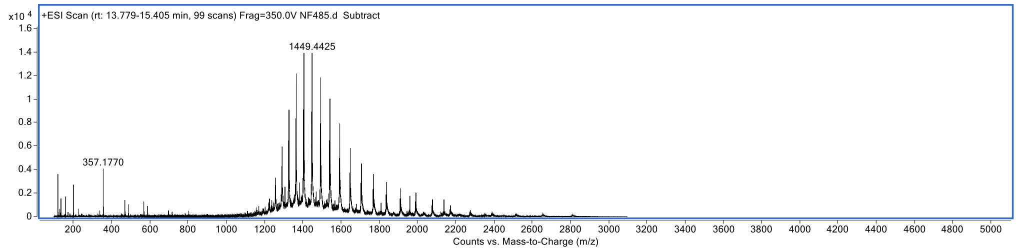

c.

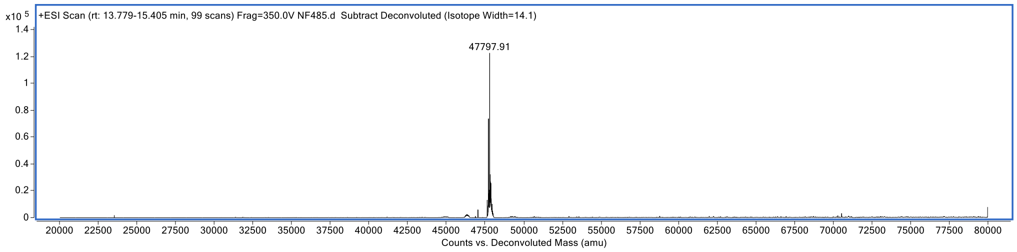

d.

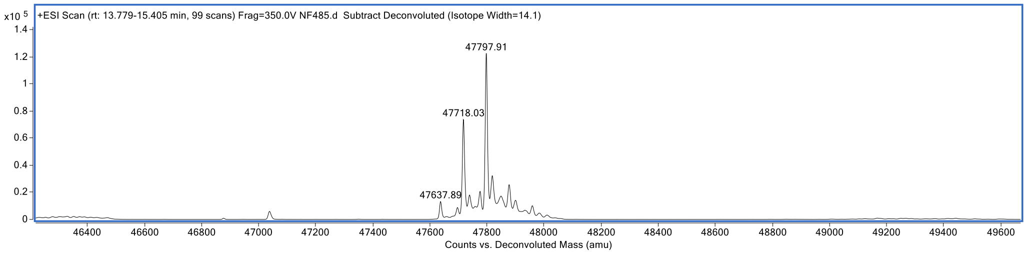

Figure S11 - LC-MS analysis of CLT conjugate **5** (acyl transfer of thioester conjugate **4** at pH 8.4, 12 ºC, 72 h); a. TIC, b. non-deconvoluted ion-series c. deconvoluted ion series mass spectrum, d. zoomed in deconvoluted ion series mass spectrum; observed mass of 47638 corresponds to native Fab, 47718 corresponds to one acyl addition (expected 47718), 47798 corresponds to two acyl additions (expected 47798).

Acyl transfer of thioester conjugate 4 under different pH/ temperature conditions

The acyl transfer of thioester conjugate **4** was examined under a variety of pH and temperature combinations. The protocol employed for all conditions was identical to the one reported on *page 27*, with the only difference being the pH of the final buffer, the temperature and time of incubation. The outcome is presented below in *Table S2* and the LC-MS data for entries 4 and 8 in *Figures S12-S13*.

Entry	pH	Temperature	Time	AAR
1	9.0	12	48 h	1.3
2	8.4	22	72 h	1.3
3	8.4	12	72 h	1.5
4	8.0	37	24 h	1.0
5	8.0	22	72 h	1.4
6	7.7	37	24 h	1.2
7	7.4	37	72 h	1.3
8	7.4	22	72 h	N/A

Table S2 - Summary of different conditions employed for the acyl transfer of thioester conjugate **4** and the AAR obtained in each case.

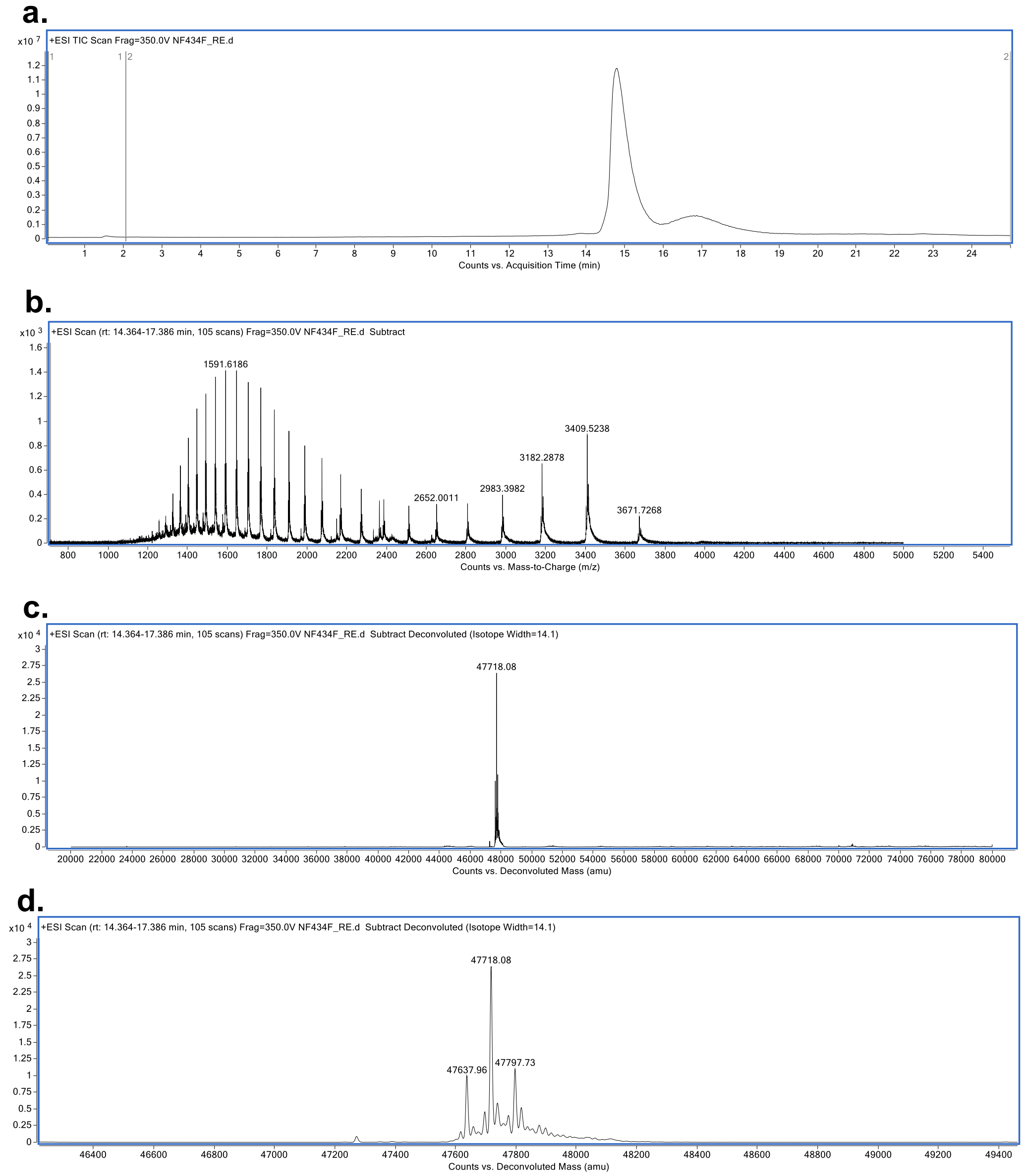

Figure S12 - LC-MS analysis of acyl transfer of thioester conjugate **4** at pH 8.0, 37 ºC, 24 h; a. TIC, b. non-deconvoluted ion-series c. deconvoluted ion series mass spectrum, d. zoomed in deconvoluted ion series mass spectrum; observed mass of 47638 corresponds to native Fab, 47718 corresponds to one acyl addition (expected 47718), 47798 corresponds to two acyl additions (expected 47798).

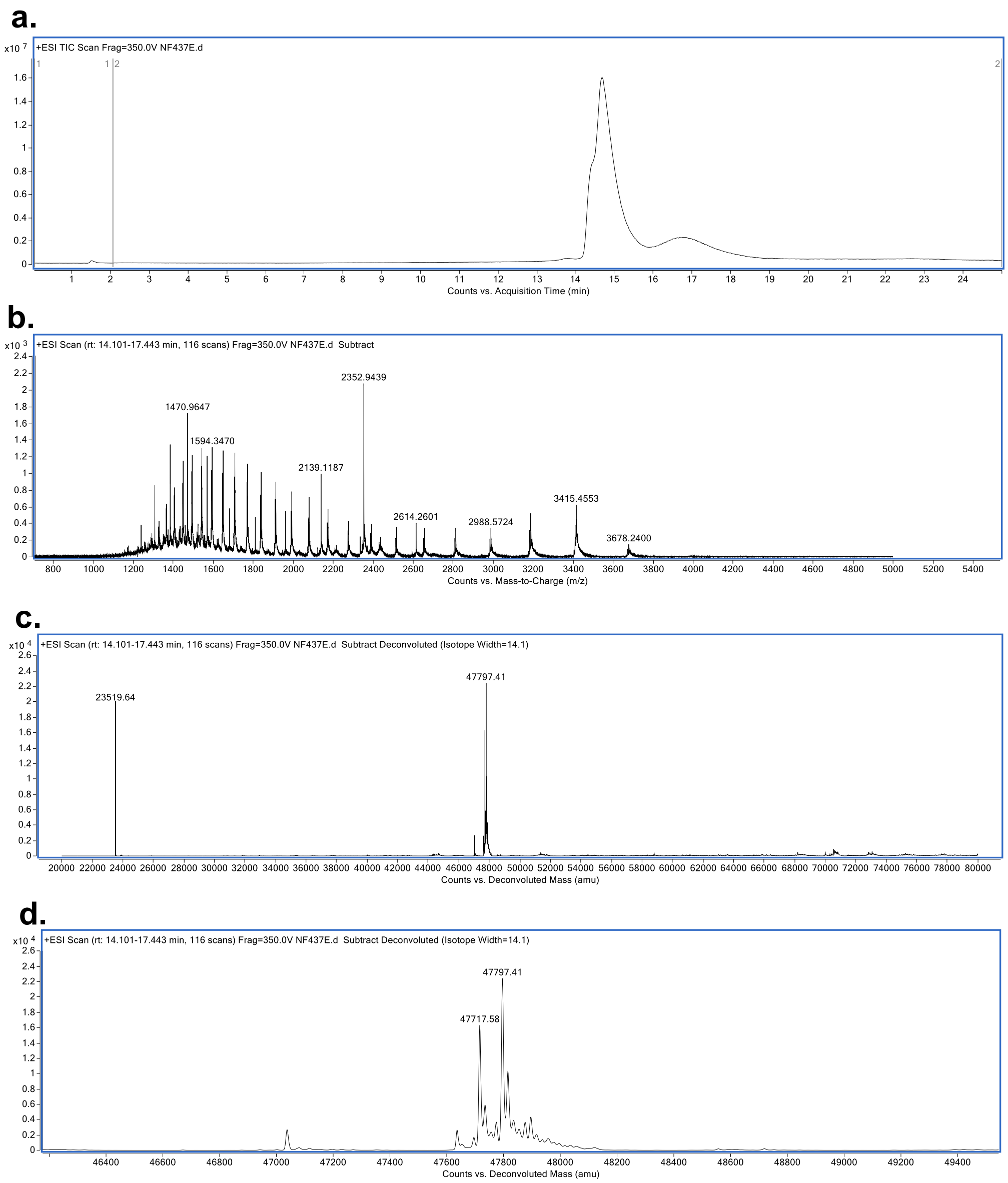

Figure S13 - LC-MS analysis of acyl transfer of thioester conjugate **4** at pH 7.4, 22 ºC, 72 h; a. TIC, b. non-deconvoluted ion-series c. deconvoluted ion series mass spectrum, d. zoomed in deconvoluted ion series mass spectrum; observed mass of 23520 corresponds to one acyl addition on LC suggesting that transfer is incomplete (expected 23520), 47718 corresponds to one acyl addition (expected 47718), 47798 corresponds to two acyl additions (expected 47798).

Treatment of CLT conjugate 5 with cysteine

To CLT conjugate **5** (50.0 μL, 0.001 μmol, 0.95 mg/mL, 20 μM) in BBS buffer (pH 8.4) was added L-Cysteine.HCl (2.5 μL, 0.100 μmol, 40 mM solution in BBS buffer (pH 8.4), 100 eq.). After 2 h at 37 ^{o}C, the excess L-Cysteine.HCl was removed *via* ultrafiltration (10 kDa MWCO) into H_2O and the sample submitted for LC-MS analysis.

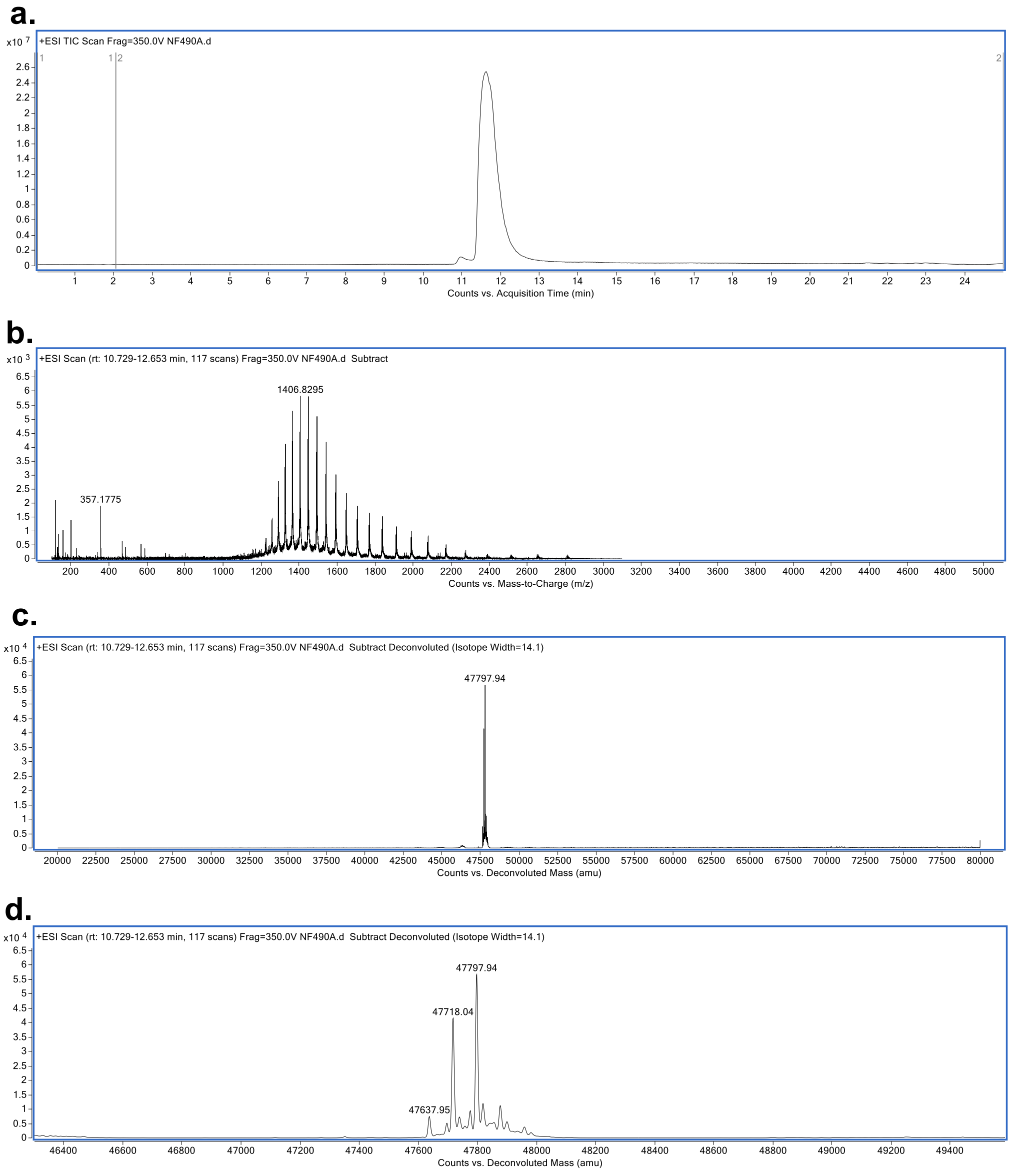

Figure S14 - LC-MS analysis of CLT conjugate **5** treated with cysteine; a. TIC, b. non-deconvoluted ion-series c. deconvoluted ion series mass spectrum, d. zoomed in deconvoluted ion series mass spectrum; observed mass of 47638 corresponds to native Fab, 47718 corresponds to one acyl addition (expected 47718), 47798 corresponds to two acyl additions (expected 47798).

TCEP reduction of CLT conjugate 5 and capping with *N*-methylmaleimide

CLT conjugate **5** was buffer exchanged to conjugation buffer *via* ultrafiltration (10 kDa MWCO). The concentration was determined by UV/Vis absorbance and adjusted to 20 μM (100 μL, 0.0020 μmol, 0.95 mg/mL). To this solution was added TCEP (1.0 μL, 0.020 μmol, 20 mM solution in H_2O, 10 eq.). After 1 h at 37 °C, *N*-methylmaleimide (0.5 μL, 0.020 μmol, 40 mM solution in DMF, 10 eq.) was added and the resultant mixture was incubated at 22 °C for 1 h. The excess maleimide was then removed *via* ultrafiltration (10 kDa MWCO) into H_2O and the sample submitted for LC-MS analysis.

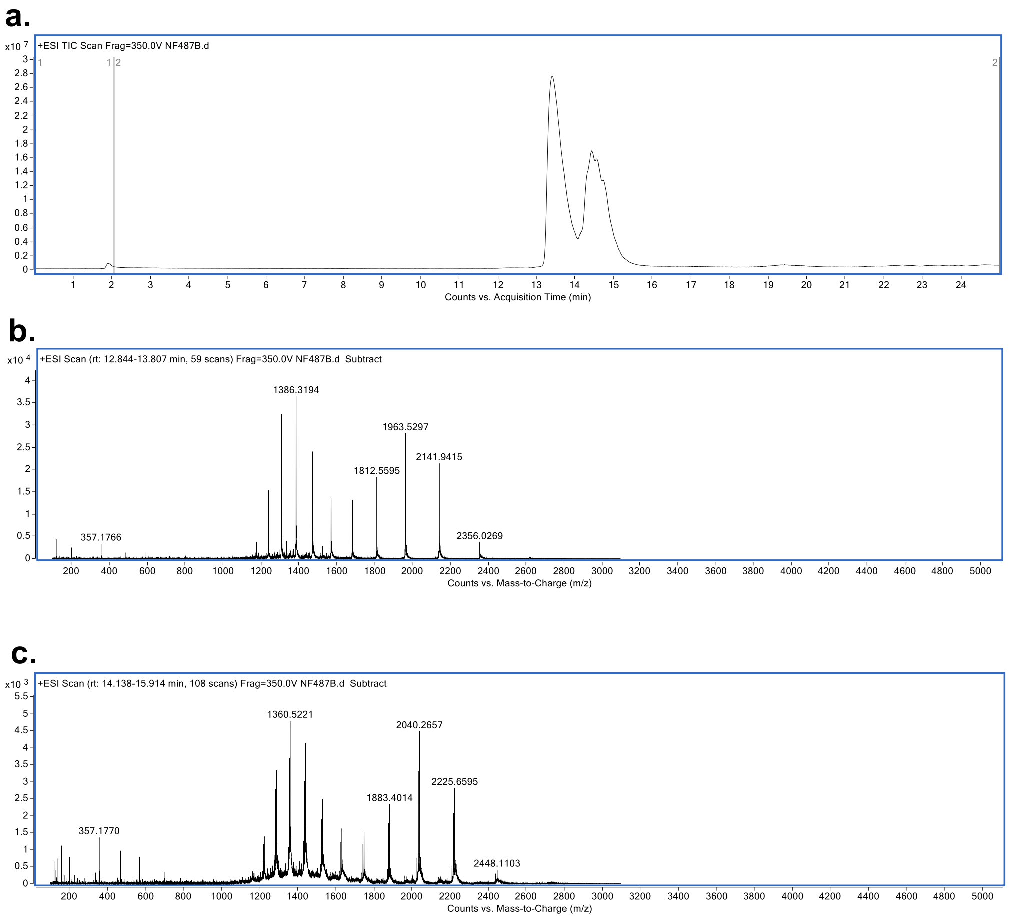

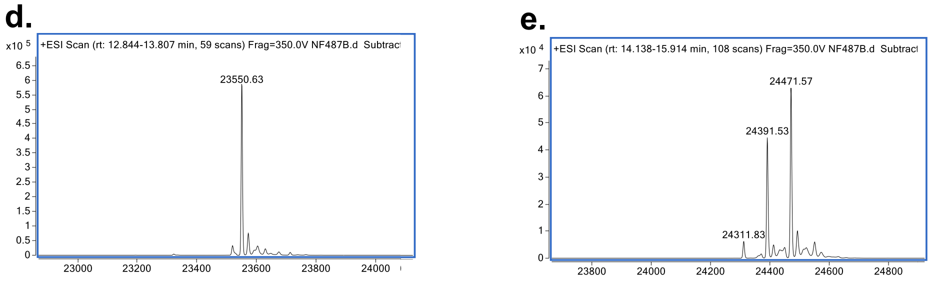

Figure S15 - LC-MS analysis of TCEP reduction and *N*-methylmaleimide capping of CLT conjugate **5**; a. TIC, b. non-deconvoluted ion-series of LC, c. non-deconvoluted ion-series of HC, d. deconvoluted ion series mass spectrum of LC; observed mass of 23550 corresponds to maleimide-capped LC (expected 23551), e. deconvoluted ion series mass spectrum of HC; observed mass of 24312 corresponds to maleimide-capped HC (expected 24312), 24392 corresponds to maleimide-capped HC containing one acyl molecule (expected 24392) , 24472 corresponds to maleimide-capped HC containing two acyl molecules (expected 24472). HC and LC were deconvoluted separately for clarity.

CuAAC of CLT conjugate 5 with AlexaFluor488 to produce functionalized conjugate 6

To a solution of CLT conjugate **5** (150 μL, 0.0030 μmol, 20 μM, 0.95 mg/mL) in BBS buffer (pH 8.4) was added THPTA ligand (1.8 μL, 0.18 μmol, 100 mM solution in H_2O), followed by $CuSO_4$ (1.8 μL, 0.036 μmol, 20 mM solution in H_2O). To this mixture was added AlexaFluor488 azide (6.0 μL, 0.060 μmol, 10 mM solution in DMF, 20 eq., Thermo Fisher Scientific), followed by sodium ascorbate (18.2 μL, 1.82 μmol, 100 mM solution in H_2O). The resultant mixture was incubated for 2 h at 37 °C. Following this period, excess reagents were removed using a desalting column (PD MiniTrap™ G-25, GE Healthcare) and repeated ultrafiltration (10 kDa MWCO) into conjugation buffer. The sample was then analyzed by SDS-PAGE (Figure S17, lanes 7 and 8). A sufficiently resolved LC-MS spectrum could not be obtained, presumably due to residual protein bound Cu(I) which interferes with the protein's charge states.[5] The fluorophore-to-antibody ratio (FAR) was determined photometrically, as described by the manufacturer. The UV/Vis absorption spectrum of the conjugate was obtained (Figure S16) and the FAR was calculated using the following formula, where *Cf* is the correction factor for the absorbance of AlexaFluor488 at 280 nm:

$$FAR = \frac{\frac{Abs_{495}}{\varepsilon_{495}}}{\frac{Abs_{280} - (Cf \cdot Abs_{495})}{\varepsilon_{280}}} = \frac{\frac{0.2582}{71{,}000}}{\frac{0.1944 - (0.11 \cdot 0.2582)}{68{,}590}} = 1.5$$

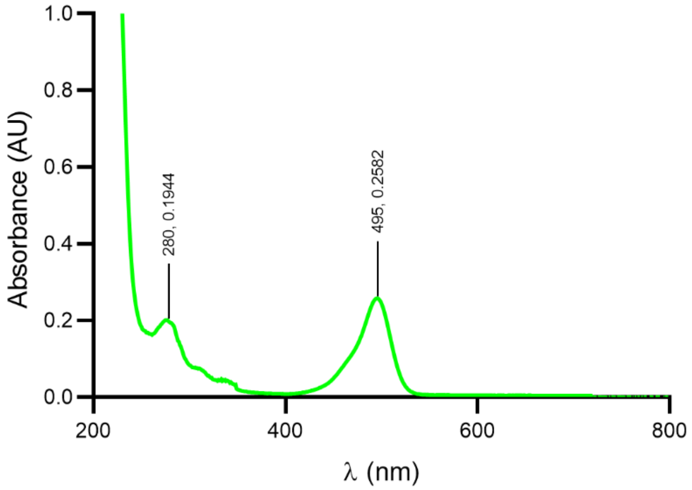

Figure S16 - UV/Vis absorbance of CLT conjugate **5** after CuAAC with AlexaFluor488.

SDS-PAGE analysis of cysteine-to-lysine transfer reaction employing MESNa thioester 3

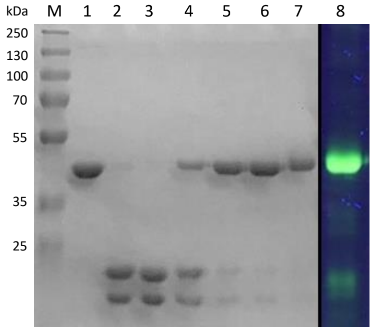

Figure S17 - M. Molecular marker, 1. Native Fab, 2. Reduced Fab, 3. Fab after transthioesterification with MESNa thioester **3**, 4. Acyl transfer (24 h), 5. Acyl transfer (48 h), 6. Acyl transfer (72 h) – CLT conjugate **5**, 7. Clicked CLT conjugate **5** with AlexaFluor488, 8. Clicked CLT conjugate **5** with AlexaFluor 488 (picture taken on a UV-transilluminator).

Reaction of anti-HER2 Fab with bis-thioester 7

To anti-HER2 Fab (50 µL, 0.0075 µmol, 150 µM, 7.15 mg/mL) in conjugation buffer was added bis-thioester **7** (5.0 µL, 0.75 µmol, 150 mM solution in DMF, 100 eq.) and the resultant solution was incubated at 22 °C for 30 min. The excess reagent was then removed *via* ultrafiltration (10 kDa MWCO) into H_2O and the sample submitted for LC-MS analysis.

a.

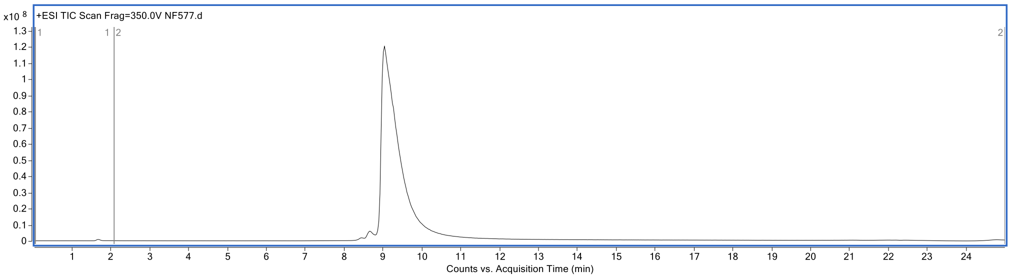

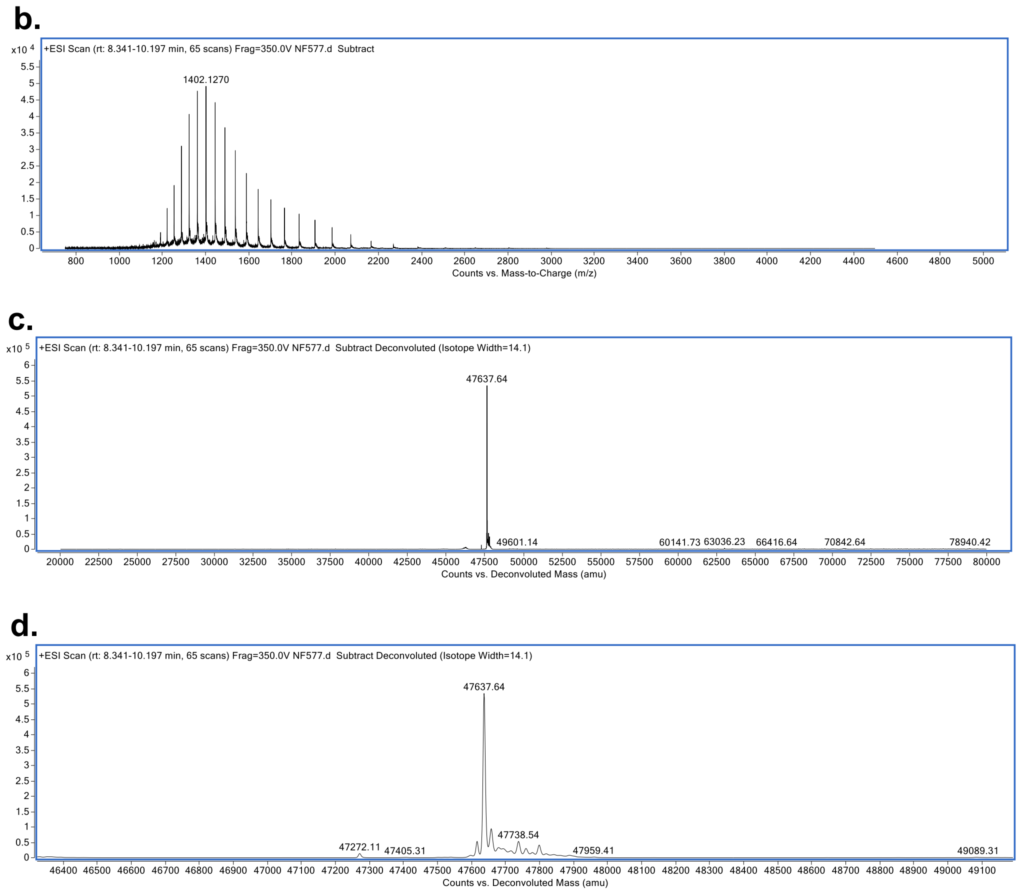

Figure S18 - LC-MS analysis of control reaction of anti-HER2 Fab with bis-thioester **7** (100 eq., 30 min, 22 °C); a. TIC, b. non-deconvoluted ion-series, c. deconvoluted ion series mass spectrum, d. zoomed in deconvoluted ion series mass spectrum; observed mass of 47638 corresponds to native Fab.

Transthioesterification of anti-HER2 Fab with bis-thioester 7 (bridged thioester conjugate 8)

To anti-HER2 Fab (40 µL, 0.0060 µmol, 150 µM, 7.15 mg/mL) in conjugation buffer was added TCEP (0.4 µL, 0.060 µmol, 150 mM solution in H_2O, 10 eq.). After 1 h at 37 °C, thioester **7** (4.0 µL, 0.60 µmol, 150 mM solution in DMF, 100 eq.) was added to the conjugation and the resultant mixture was incubated at 22 °C for 30 min. The excess thioester was then removed *via* ultrafiltration (10 kDa MWCO) into H_2O and the sample submitted for LC-MS analysis, where it was kept at 4 °C until analyzed.

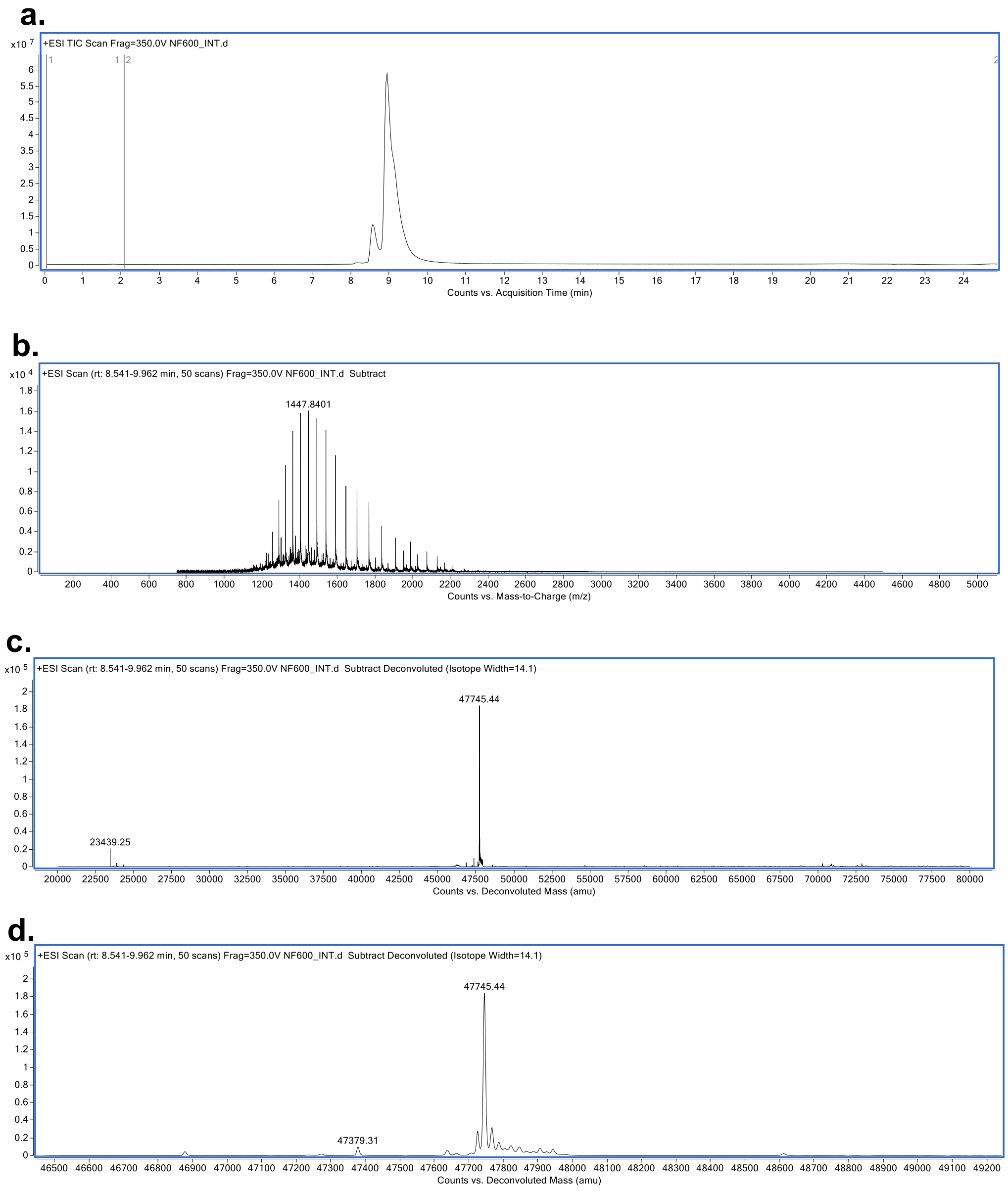

Figure S19 - LC-MS analysis of bridged thioester conjugate **8** (transthioesterification of anti-HER2 Fab with bis-thioester **7** (100 eq., 30 min, 22 ^{o}C)); a. TIC, b. non-deconvoluted ion-series c. deconvoluted ion series mass spectrum, d. zoomed in deconvoluted ion series mass spectrum; observed mass of 47745 corresponds to Fab bridged with thioester **7** (expected 47745).

Acyl transfer of bridged thioester conjugate 8 at pH 8.4, 12 ºC, 48 h

To anti-HER2 Fab (60 µL, 0.009 µmol, 150 µM, 7.15 mg/mL) in conjugation buffer was added TCEP (0.6 µL, 0.09 µmol, 150 mM solution in H_2O, 10 eq.). After 1 h at 37 ^oC, thioester **7** (6.0 µL, 0.9 µmol, 150 mM solution in DMF, 100 eq.) was added in the conjugation and the resultant mixture was incubated at 22 ^oC for 30 min. The excess thioester was then removed *via* ultrafiltration (10 kDa MWCO) into conjugation buffer and buffer exchanged into BBS buffer (pH 8.4), *via* ultrafiltration (10 kDa MWCO). The concentration was determined by UV/Vis absorbance and adjusted to 20 µM (400 µL, 0.95 mg/mL). The resultant solution was incubated for 48 h at 12 ^oC. BME (40.0 µL, 0.8 µmol, 20 mM solution in BBS, 100 eq.) was then added. After 2 h at 37 ^oC, the excess BME was removed *via* ultrafiltration (10 kDa MWCO) into H_2O and the sample submitted for LC-MS analysis.

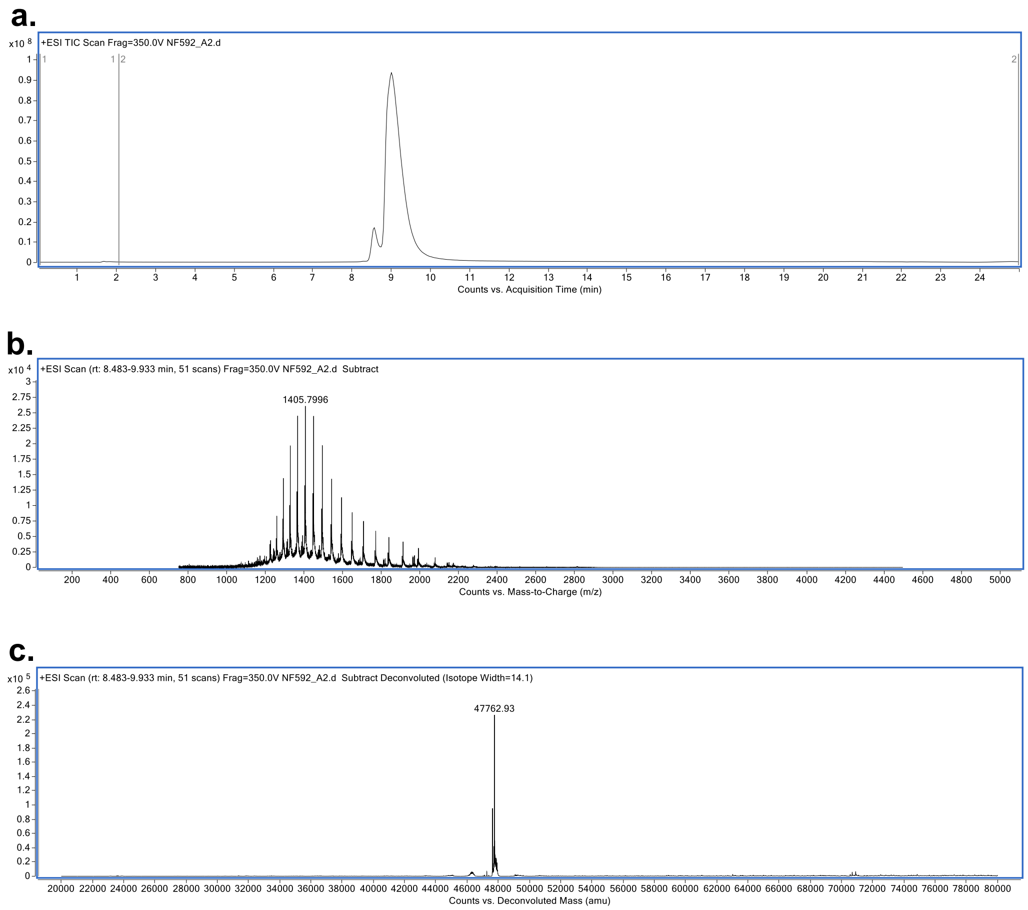

d.

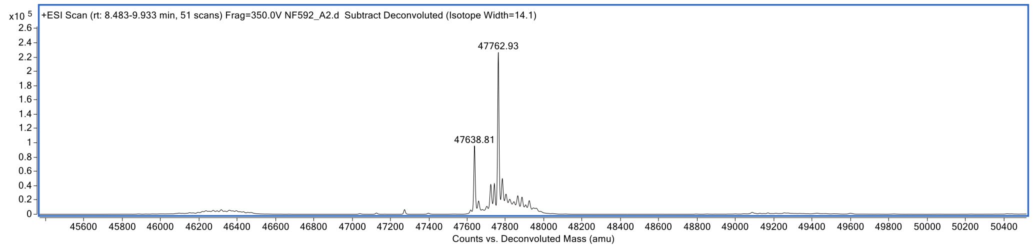

Figure S20 - LC-MS analysis of acyl transfer of bridged thioester conjugate **8** at pH 8.4, 12 ^{o}C, 48 h; a. TIC, b. non-deconvoluted ion-series, c. deconvoluted ion series mass spectrum, d. zoomed in deconvoluted ion series mass spectrum; observed mass of 47639 corresponds to native Fab, 47763 corresponds to Fab with one acyl addition of hydrolyzed thioester **7** (expected 47762).

Acyl transfer of bridged thioester conjugate 8 at pH 8.4, 37 ^{o}C, 6 h (CLT conjugate 9)

To anti-HER2 Fab (400 μL, 0.0600 μmol, 150 μM, 7.15 mg/mL) in conjugation buffer was added TCEP (4.0 μL, 0.60 μmol, 150 mM solution in H_2O, 10 eq.). After 1 h at 37 ^{o}C, thioester **7** (40.0 μL, 6.0 μmol, 150 mM solution in DMF, 100 eq.) was added in the conjugation and the resultant mixture was incubated at 22 ^{o}C for 30 min. The excess thioester was then removed *via* ultrafiltration (10 kDa MWCO) into conjugation buffer and buffer exchanged into BBS buffer (pH 8.4), *via* ultrafiltration (10 kDa MWCO). The concentration was determined by UV/Vis absorbance and adjusted to 20 μM (2400 μL, 0.95 mg/mL). The resultant solution was incubated for 6 h at 37 ^{o}C. BME (480 μL, 9.60 μmol, 20 mM solution in BBS, 200 eq.) was then added. After 2 h at 37 ^{o}C, the excess BME was removed *via* ultrafiltration (10 kDa MWCO) into H_2O and the sample submitted for LC-MS analysis.

a.

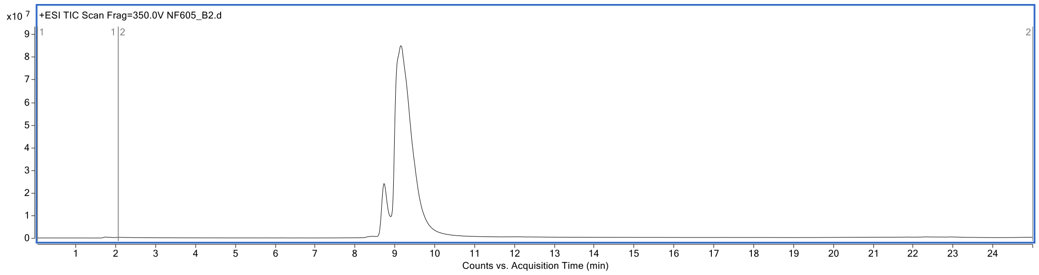

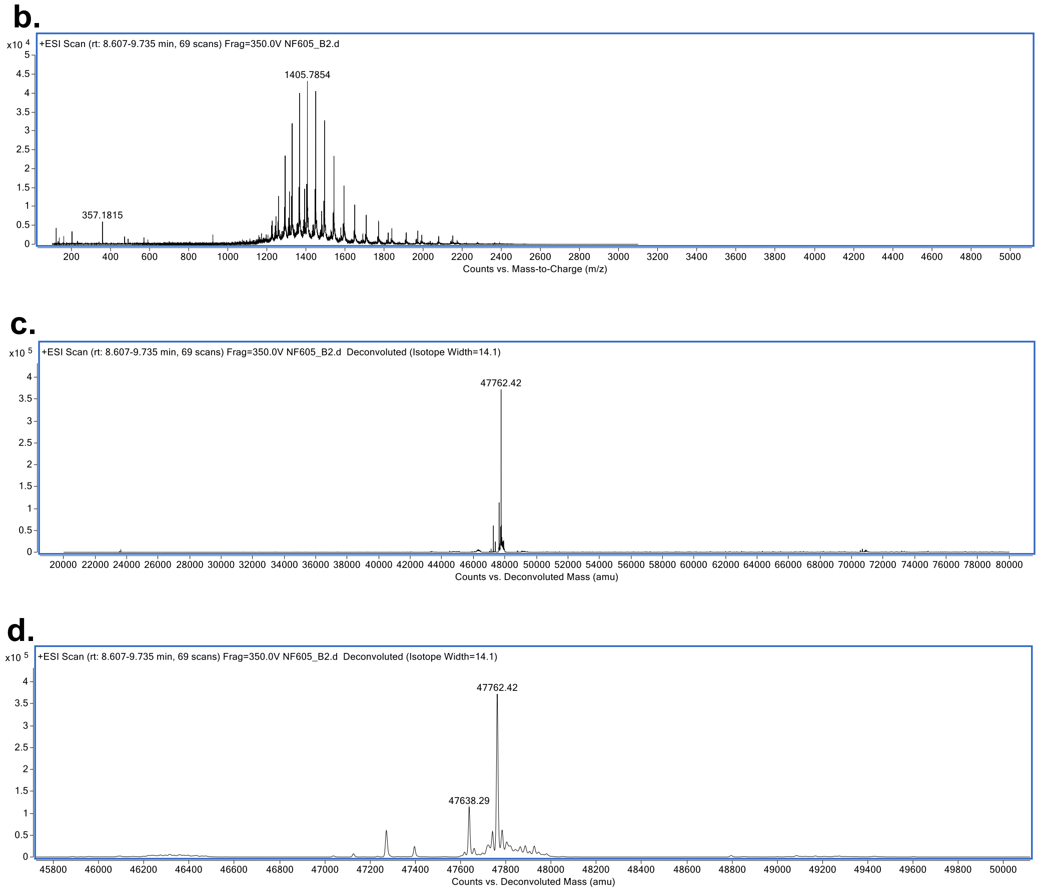

Figure S21 - LC-MS analysis of CLT conjugate **9** (acyl transfer of bridged thioester **8** at pH 8.4, 37 °C, 6 h); a. TIC, b. non-deconvoluted ion-series, c. deconvoluted ion series mass spectrum, d. zoomed in deconvoluted ion series mass spectrum; observed mass of 47638 corresponds to native Fab, 47761 corresponds to Fab with one acyl addition of hydrolyzed thioester **7** (expected 47762).

TCEP reduction of CLT conjugate 9 and capping with *N*-methylmaleimide

CLT conjugate **9** was buffer exchanged to conjugation buffer *via* ultrafiltration (10 kDa MWCO). The concentration was determined by UV/Vis absorbance and adjusted to 20 μM (100 μL, 0.0020 μmol, 0.95 mg/mL). To this solution was added TCEP (1.0 μL, 0.020 μmol, 20 mM solution in H_2O, 10 eq.). After 1 h at 37 °C, *N*-methylmaleimide (0.5 μL, 0.020 μmol, 40 mM solution in DMF, 10 eq.) was added and the resultant mixture was incubated at 22 °C for 1 h. The excess maleimide was then removed *via* ultrafiltration (10 kDa MWCO) into H_2O and the sample submitted for LC-MS analysis.

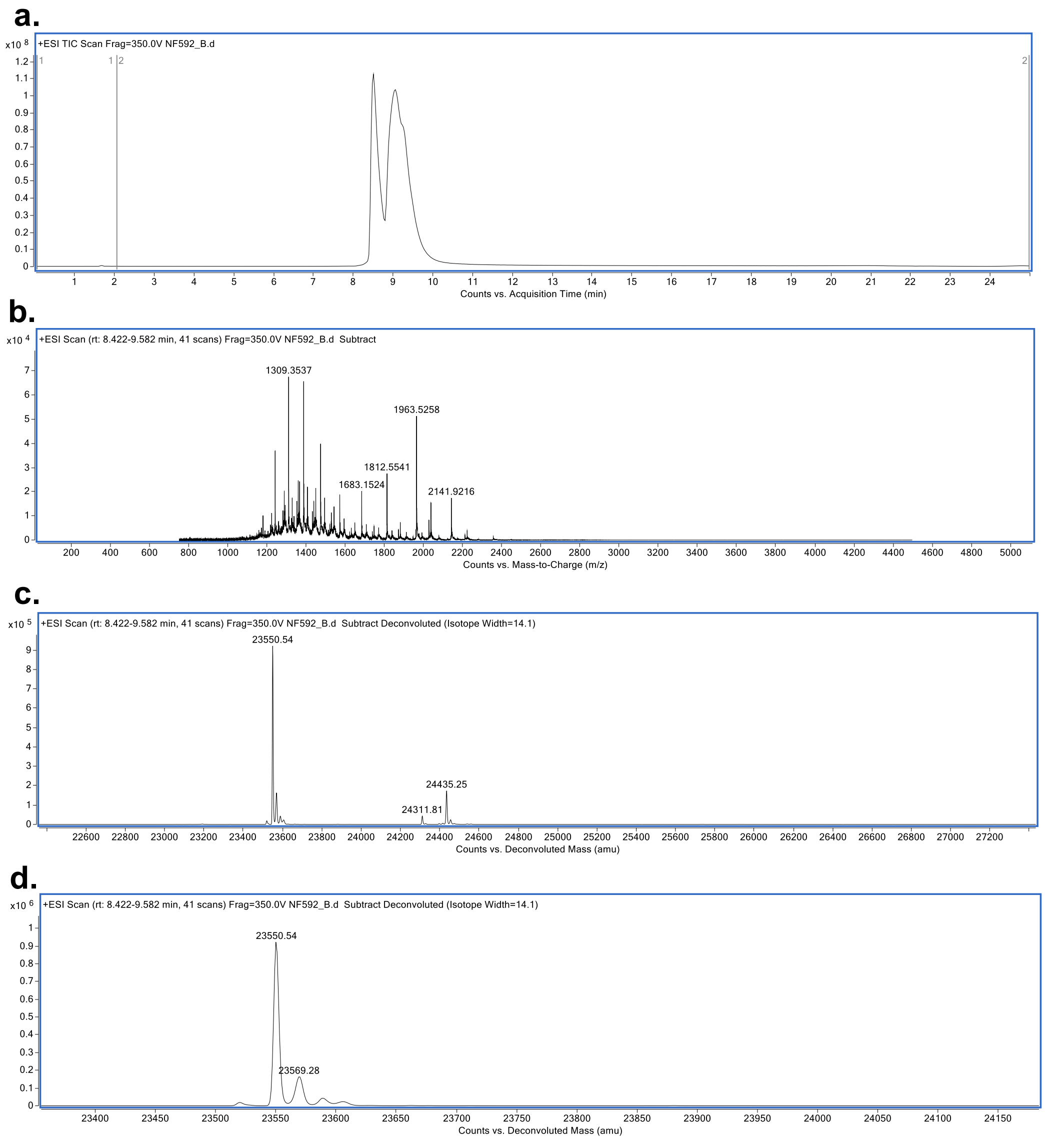

Figure S22 - LC-MS analysis of TCEP reduction and *N*-methylmaleimide capping of CLT conjugate **9**; a. TIC, b. non-deconvoluted ion-series c. deconvoluted ion series mass spectrum; observed mass of 23551 corresponds to maleimide-capped LC (expected 23551), 24312 corresponds to maleimide-capped HC (expected 24312), 24435 corresponds to maleimide-capped HC containing one acyl molecule (expected 24437), d. zoomed in deconvoluted spectrum at LC showing a second peak at 23569 corresponding to hydrolysed maleimide-capped LC (expected 23569).

CuAAC of CLT conjugate 9 with AlexaFluor488 to produce functionalized conjugate 10

To a solution of CLT conjugate **9** (150 µL, 0.0030 µmol, 20 µM, 0.95 mg/mL) in BBS buffer (pH 8.0) was added THPTA ligand (1.8 µL, 0.18 µmol, 100 mM solution in H_2O), followed by $CuSO_4$ (1.8 µL, 0.036 µmol, 20 mM solution in H_2O). To this mixture was added AlexaFluor488 azide (6.0 µL, 0.060 µmol, 10 mM solution in DMF, 20 eq., Thermo Fisher Scientific), followed by sodium ascorbate (18.2 µL, 1.82 µmol, 100 mM solution in H_2O). The resultant mixture was incubated for 2 h at 37 °C. Following this period, excess reagents were removed using a desalting column (PD MiniTrap™ G-25, GE Healthcare) and repeated ultrafiltration (10 kDa MWCO) into conjugation buffer. The sample was then analyzed by LC-MS (Figure S24) and SDS-PAGE (Figure S25, lanes 5 and 6). The fluorophore-to-antibody ratio (FAR) was determined photometrically, as described by the manufacturer. The UV/Vis absorption spectrum of the conjugate was obtained (Figure S23) and the FAR was calculated using the following formula, where *Cf* is the correction factor for the absorbance of AlexaFluor488 at 280 nm:

$$FAR = \frac{\frac{Abs_{495}}{\varepsilon_{495}}}{\frac{Abs_{280} - (Cf \cdot Abs_{495})}{\varepsilon_{280}}} = \frac{\frac{0.5997}{71{,}000}}{\frac{0.8275 - (0.11 \cdot 0.5997)}{68{,}590}} = 0.8$$

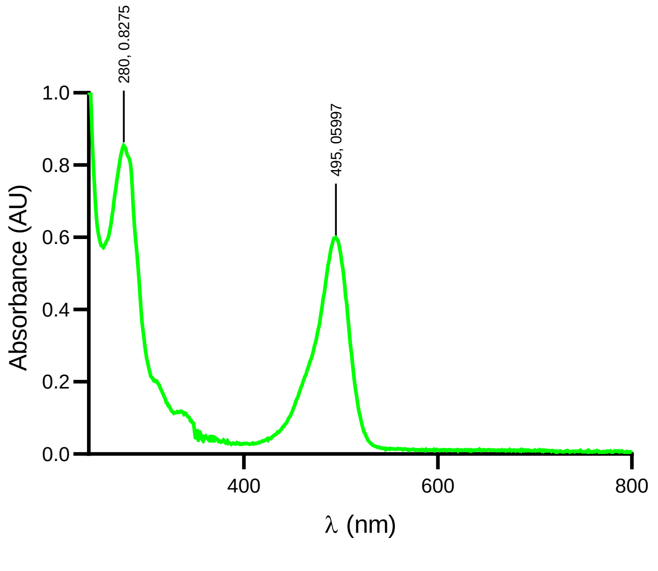

Figure S23 - UV/Vis absorbance of CLT conjugate **9** after CuAAC with AlexaFluor488.

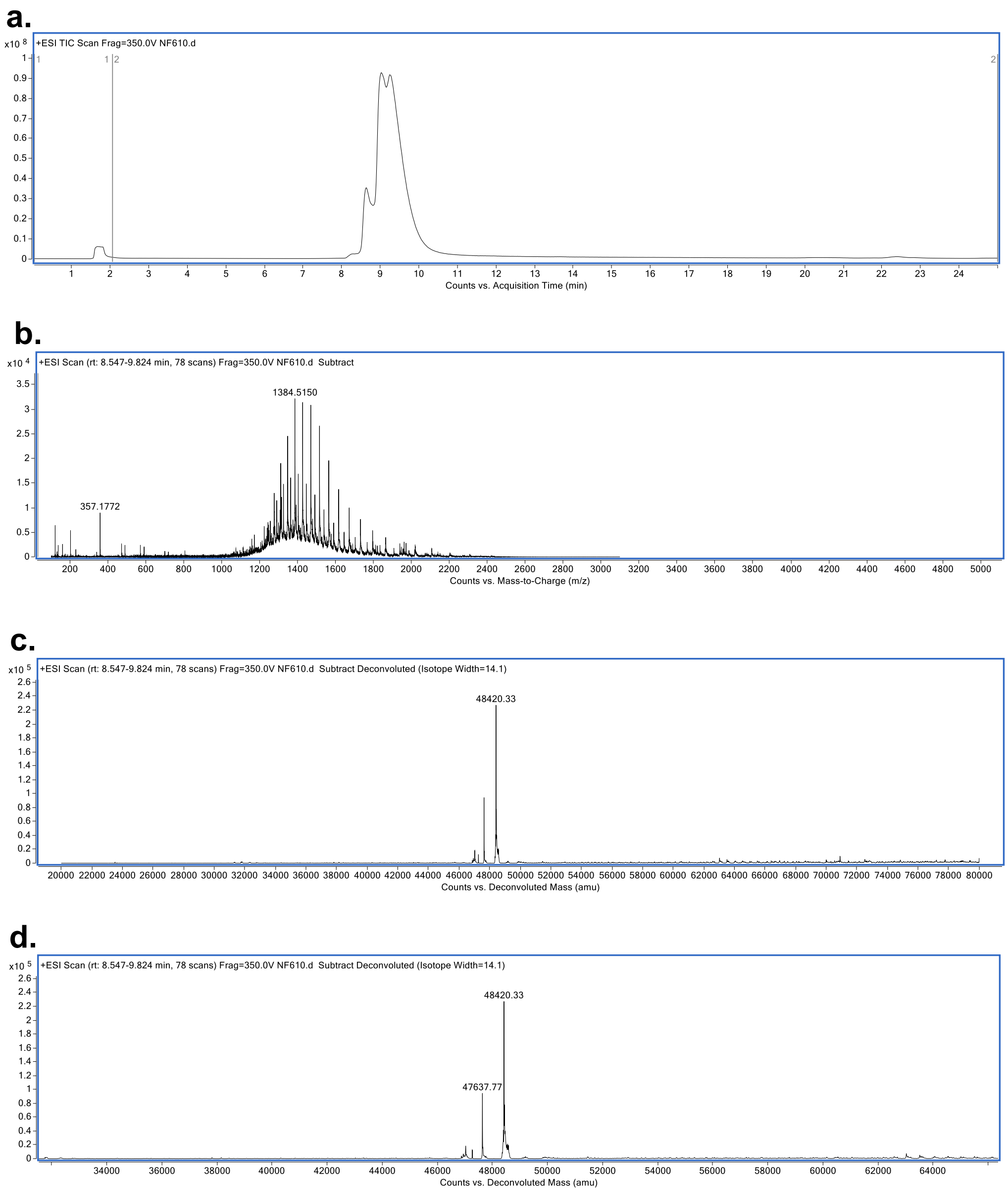

Figure S24 - LC-MS analysis of CLT conjugate **9** after CuAAC with AlexaFluor488; a. TIC, b. non-deconvoluted ion-series c. deconvoluted ion series mass spectrum, d. zoomed in deconvoluted ion series mass spectrum; observed mass of 47638 corresponds to native Fab, 48420 corresponds to one acyl addition clicked with AlexaFluor488 (expected 48420).

SDS-PAGE analysis of cysteine-to-lysine transfer reaction employing bis-thioester 7

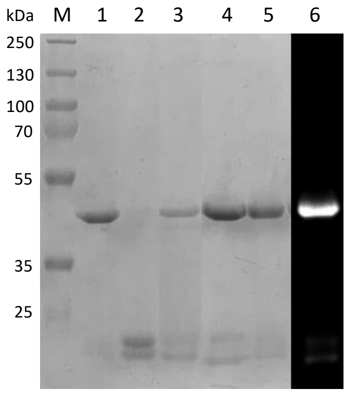

Figure S25 - M. Molecular marker, 1. Native Fab, 2. Reduced Fab, 3. Fab bridged with thioester **7**, 4. CLT conjugate **9**, 5. Clicked CLT conjugate **9** with AlexaFluor488, 6. clicked CLT conjugate **9** with AlexaFluor488 (picture taken on a UV-transilluminator).

Size Exclusion Chromatography (SEC)

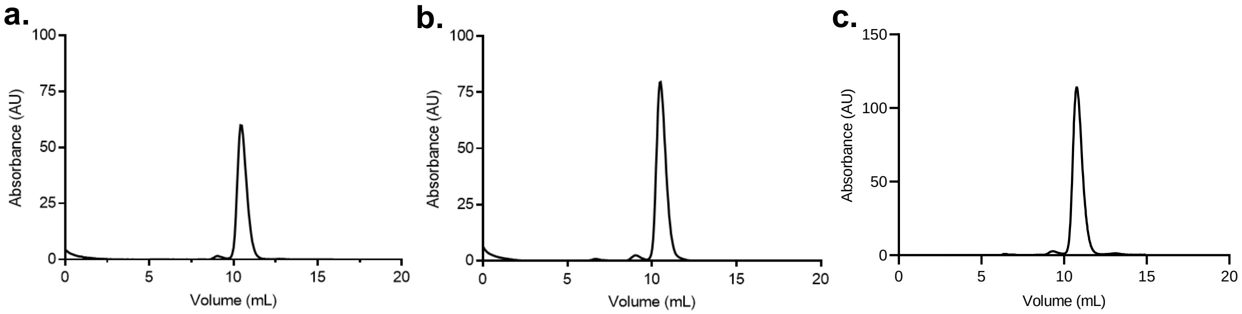

Figure S26 - Size Exclusion Chromatograms of a. native Fab, b. CLT conjugate **5**, c. CLT conjugate **9**; SEC conditions: 24 mL Superdex™ 200 10/300 GL, GE Healthcare, sample volume 200 μL, loop volume 1 mL, flowrate 0.25 mL/min.

Enzyme-Linked Immunosorbent Assay (ELISA)

Protocol for HER2 ELISA

A 96-well plate was coated for 1 h at RT with HER2 (Sino Biological, 100 µL/well, 0.25 µg/mL solution in PBS). After washing (3 × 0.1% Tween® 20 in PBS, followed by 3 × PBS), the wells were blocked for 1 h at RT with 5% Marvel milk powder (Premier foods) in PBS (200 µL/well). The wells were then washed and the following dilutions of native Fab and CLT conjugate **5** or **9** were applied: 270 nM, 90 nM, 30 nM, 10 nM, 3.33 nM, 1.11 nM, 0.37 nM, 0.123 nM, 0.0412 nM, 0.0137 nM, prepared in 1% Marvel solution in 0.1% Tween® 20 in PBS (100 µL/well). The assay was then incubated at RT for 1 h, washed and the detection antibody (Anti-Human IgG, Fab specific-HRP antibody, Sigma Aldrich, 1:5000 in 1% Marvel solution in 0.1% Tween® 20 in PBS) was added (100 µL/well). After 1 h at RT, the plates were washed and *o*-phenylenediamine dihydrochloride (Sigma-Aldrich, 100 µL/well, 0.5 mg/mL in a phosphate-citrate buffer with sodium perborate) was added. Once a yellow-orange colour was observed, the reaction was stopped by addition of HCl (4M, 50 µL/well). Absorbance was immediately measured at 450 nm and was corrected by subtracting the average of negative controls (i.e. PBS had been added to some of the wells instead of HER2 or instead of the samples). Each sample was tested in triplicate and errors are shown as the standard deviation of the average. ELISA data was analyzed with Graphpad Prism 7.03 and the values have been normalized.

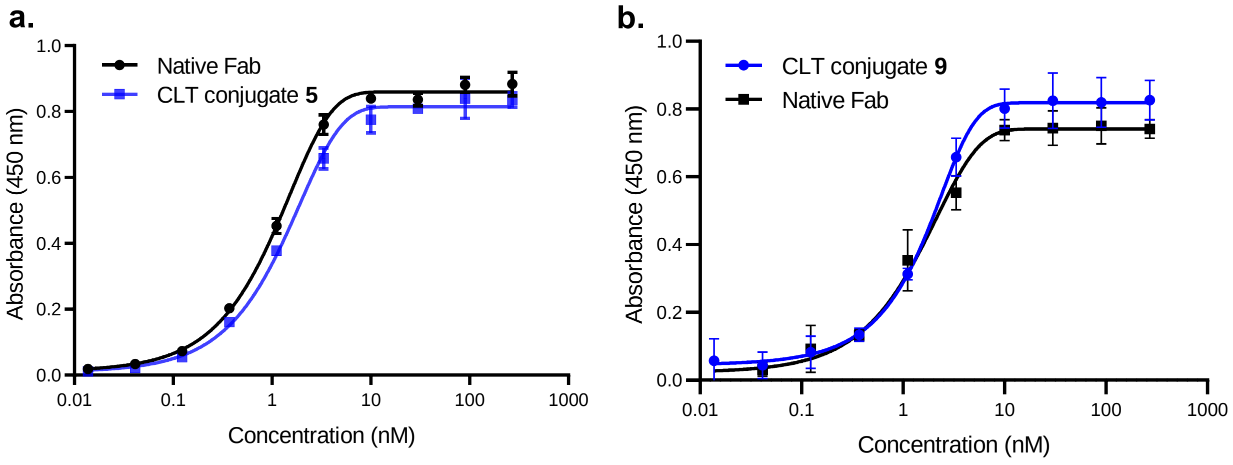

Figure S27 - ELISA analysis of a. CLT conjugate **5** and native Fab, b. CLT conjugate **9** and native Fab against HER2.

Trypsin digestion of CLT conjugate 5

CLT conjugate **5** was buffer exchanged into H_2O (10 kDa MWCO). The concentration was determined by UV/Vis absorbance and adjusted to 450 μM (21.4 mg/mL). 14 μL of this solution were diluted to 70 μL with a solution of 6M Guanidine.HCl and 2 mM EDTA. DTT (3 μL, 100 mM solution in 100 mM Tris buffer, pH 8.0) was then added. After 75 min at 37 ^{o}C, iodoacetamide (6 μL, 100 mM solution in 100 mM Tris buffer, pH 8.0) was added and the mixture was incubated for further 75 min at 37 ^{o}C, in the dark. The reaction was diluted with 280 μL H_2O and 70 μL Tris buffer (50 mM, pH 8.0), before trypsin (Pierce^{TM} Trypsin Protease, MS Grade, 3 μL, 1 mg/mL solution in 10 mM HCl) was added. The resultant mixture was incubated for 16 h at 37 ^{o}C with shaking (300 rpm). After this period, the reaction was centrifuged and stopped by the addition of TFA (2.2 μL). The peptide mixture was then purified using a C18 cartridge (Sep-Pak C18, 360 mg sorbent); the sample was firstly washed with 1.5 mL of H_2O, 0.1% formic acid solution and eluted with 3.0 mL of 30% H_2O, 70% MeCN, 0.1% formic acid solution. It was then concentrated using a Speedvac concentrator and analysed by LC-MS and LC-MS/MS.

Sequence coverage of CLT conjugate 5 by trypsin digestion

100% sequence coverage was obtained. Three modifications were observed at K136, K221 and K225 on the heavy chain. Two modifications at K126 and K190 on the light chain were identified by the Spectrum Mill software, but the intensity of these peptides was very low and the quality of LC-MS/MS spectra was poor. This, in combination with the fact that no modification was observed in the intact LC-MS of light chain (Figure S15) led us to conclude that these modifications are artefacts or of very small amounts.

1	DIQMTQSPSS	LSASVGDRVT	IT**C**RASQDVN	TAVAWYQQKP	GKAPKLLIYS	ASFLYSGVPS	RFSGSRSGTD	FTLTISSLQP	80
81	EDFATYY**C**QQ	HYTTPPTFGQ	GTKVEIKRTV	AAPSVFIFPP	SDEQLKSGTA	SVV**C**LLNNFY	PREAKVQWKV	DNALQSGNSQ	160
161	ESVTEQDSKD	STYSLSSTLT	LSKADYEKHK	VYA**C**EVTHQG	LSSPVTKSFN	RGE**C**			214

The matched peptides cover **100%** (214/214 AA's) of the protein.

Protein Name: **Light_chain**
Species: **HUMAN**
SwissProt.herceptin Accession #: **1**
MS Digest Index #: **1**
Masses are:
pI of Protein: **7.76**
Protein MW: **23443.2** Da
Amino Acid Composition: **A14 C5 D10 E9 F9 G11 H3 I6 K13 L14 M1 N6 P12 Q15 R7 S31 T20 V16 W2 Y10**

1	EVQLVESGGG	LVQPGGSLRL	S**C**AASGFNIK	DTYIHWVRQA	PGKGLEWVAR	IYPTNGYTRY	ADSVKGRFTI	SADTSKNTAY	80
81	LQMNSLRAED	TAVYY**C**SRWG	GDGFYAMDYW	GQGTLVTVSS	ASTKGPSVFP	LAPSSKSTSG	GTAALG**C**LVK	DYFPEPVTVS	160
161	WNSGALTSGV	HTFPAVLQSS	GLYSLSSVVT	VPSSSLGTQT	YI**C**NVNHKPS	NTKVDKKVEP	KS**C**DKTH		227

The matched peptides cover **100%** (227/227 AA's) of the protein.

Protein Name: **Heavy_chain**
Species: **HUMAN**
SwissProt.herceptin Accession #: **2**
MS Digest Index #: **2**
Masses are:
pI of Protein: **8.90**
Protein MW: **24204.3** Da
Amino Acid Composition: **A16 C5 D9 E6 F6 G23 H4 I5 K13 L16 M2 N8 P12 Q7 R7 S30 T20 V21 W5 Y12**

MS Digest - Agilent Spectrum Mill Rev. 3.3.084

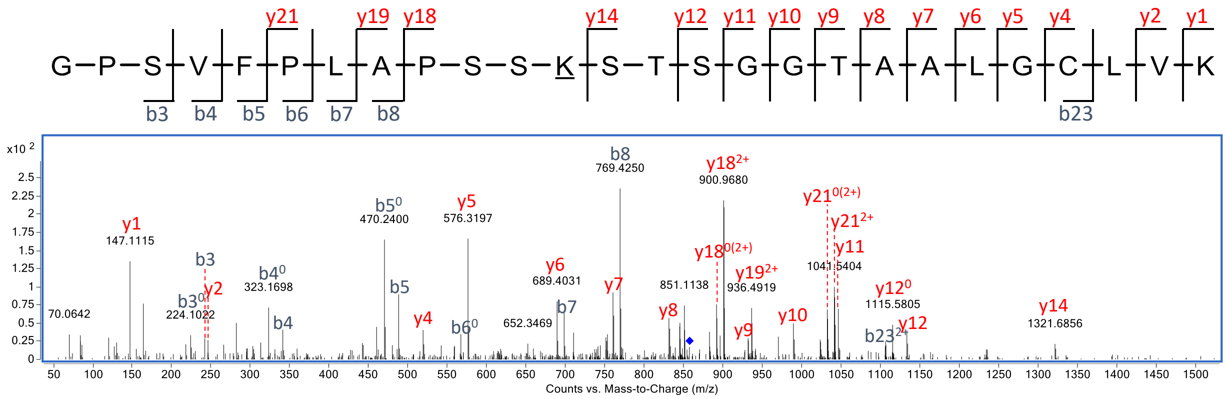

Figure S28 - LC-MS/MS spectrum of the Lys-136 modified peptide, obtained by selecting *m/z* 857.1246 [z = 3] as the precursor ion for CID; 2+ = bivalent ion (m/2), ^{0} = m-H2O, * = m-NH_3.

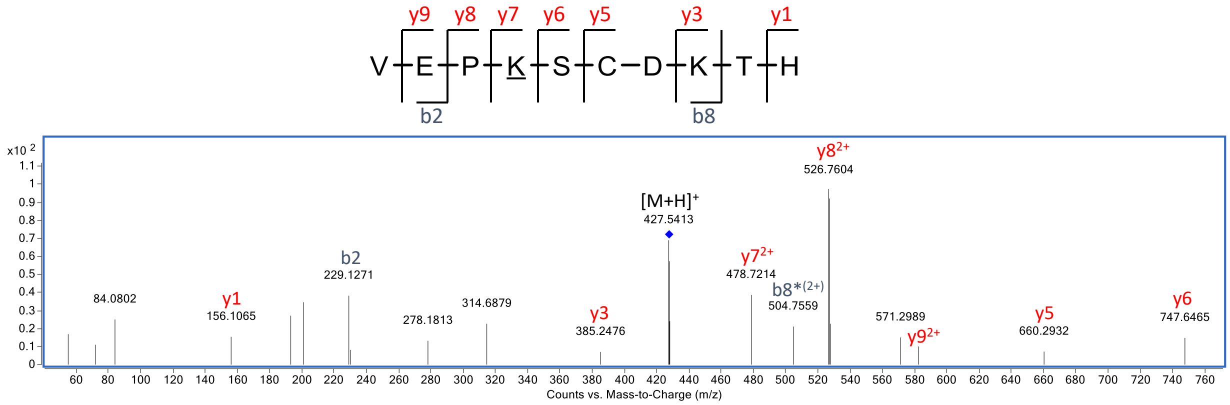

Figure S29 - LC-MS/MS spectrum of the Lys-221 modified peptide, obtained by selecting *m/z* 427.5353 [z = 3] as the precursor ion for CID; 2+ = bivalent ion (m/2).

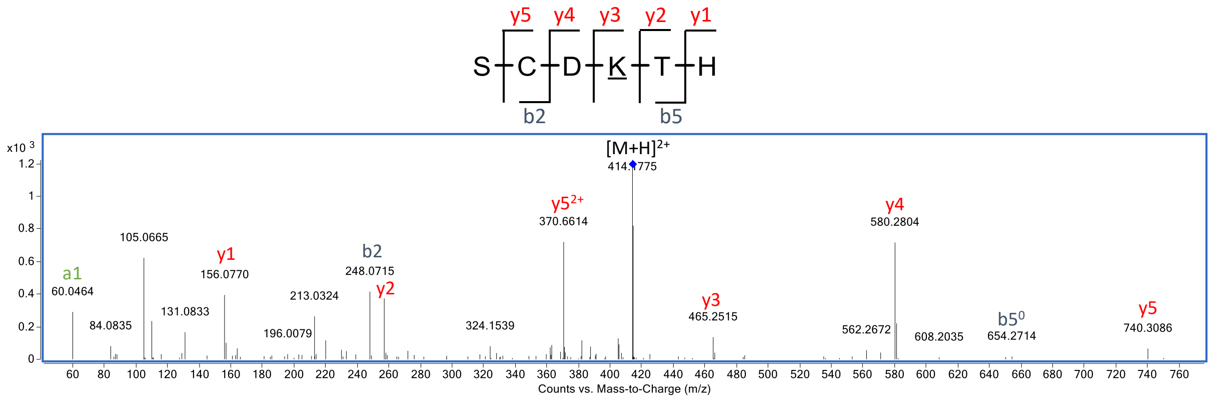

Figure S30 - LC-MS/MS spectrum of the Lys-225 modified peptide, obtained by selecting *m/z* 414.1742 [z = 2] as the precursor ion for CID; 2+ = bivalent ion (m/2).

Chymotrypsin digestion of CLT conjugate 9

CLT conjugate **9** was buffer exchanged into H_2O (10 kDa MWCO). The concentration was determined by UV/Vis absorbance and adjusted to 450 μM (21.4 mg/mL). 12 μL of this solution were diluted to 60 μL with a solution of 6M Guanidine.HCl and 2 mM EDTA. DTT (2.6 μL, 100 mM solution in 100 mM Tris buffer, pH 8.0) was then added. After 60 min at 37 ^{o}C, iodoacetamide (5.2 μL, 100 mM solution in 100 mM Tris buffer, pH 8.0) was added and the mixture was incubated for further 75 min at 37 ^{o}C, in the dark. The reaction was diluted with 240 μL H_2O and 60 μL Tris buffer (50 mM, pH 8.0), before chymotrypsin (Promega Chymotrypsin, sequencing grade, 2.6 μL, 1 mg/mL solution in 1 mM HCl) was added. The resultant mixture was incubated for 14 h at 22 ^{o}C with shaking (300 rpm). After this period, the reaction was centrifuged and stopped by the addition of TFA (1.9 μL). The peptide mixture was then purified using a C18 cartridge (Sep-Pak C18, 360 mg sorbent); the sample was firstly washed with 1.5 mL of H_2O, 0.1% formic acid solution and eluted with 3.0 mL of 30% H_2O, 70% MeCN, 0.1% formic acid solution. It was then concentrated using a Speedvac concentrator and analyzed by LC-MS and LC-MS/MS.

Sequence coverage of CLT conjugate 9 by chymotrypsin digestion

95% sequence coverage was obtained, with 100% coverage of lysine residues. Only a single modification was observed at K136 on the heavy chain.

1	DIQMTQSPSS	LSASVGDRVT	IT**C**RASQDVN	TAVAWYQQKP	GKAPKLLIYS	ASFLYSGVPS	RFSGSRSGTD	FTLTISSLQP	80
81	EDFATYY**C**QQ	HYTTPPTFGQ	GTKVEIKRTV	AAPSVFIFPP	SDEQLKSGTA	SVV**C**LLNNFY	PREAKVQWKV	DNALQSGNSQ	160
161	ESVTEQDSKD	STYSLSSTLT	LSKADYEKHK	VYA**C**EVTHQG	LSSPVTKSFN	RGE**C**			214

The matched peptides cover **98%** (211/214 AA's) of the protein.

Protein Name: **Light**
Species: **HUMAN**
SwissProt.herceptin Accession #: **1**
MS Digest Index #: **1**
Masses are:
pI of Protein: **7.76**
Protein MW: **23443.2** Da
Amino Acid Composition: **A14 C5 D10 E9 F9 G11 H3 I6 K13 L14 M1 N6 P12 Q15 R7 S31 T20 V16 W2 Y10**

1	EVQLVESGGG	LVQPGGSLRL	S**C**AASGFNIK	DTYIHWVRQA	PGKGLEWVAR	IYPTNGYTRY	ADSVKGRFTI	SADTSKNTAY	80
81	LQMNSLRAED	TAVYY**C**SRWG	GDGFYAMDYW	GQGTLVTVSS	ASTKGPSVFP	LAPSSKSTSG	GTAALG**C**LVK	DYFPEPVTVS	160
161	WNSGALTSGV	HTFPAVLQSS	GLYSLSSVVT	VPSSSLGTQT	YI**C**NVNHKPS	NTKVDKKVEP	KS**C**DKTH		227

The matched peptides cover **92%** (210/227 AA's) of the protein.

Protein Name: **Heavy**
Species: **HUMAN**
SwissProt.herceptin Accession #: **2**
MS Digest Index #: **2**
Masses are:
pI of Protein: **8.90**
Protein MW: **24204.3** Da
Amino Acid Composition: **A16 C5 D9 E6 F6 G23 H4 I5 K13 L16 M2 N8 P12 Q7 R7 S30 T20 V21 W5 Y12**

MS Digest - Agilent Spectrum Mill Rev. 3.3.084

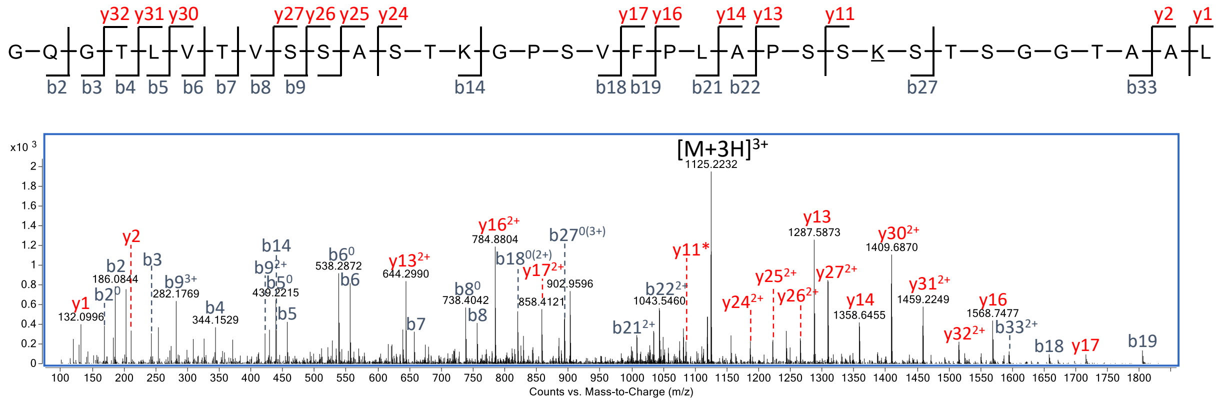

Figure S31 - LC-MS/MS spectrum of the Lys-136 modified peptide, obtained by selecting *m/z* 1124.9054 [z = 3] as the precursor ion for CID; 2+ = bivalent ion (m/2), 3+ trivalent ion (m/3).

Overlaid Fab crystal structures derived from PDB files 1HZH and 6BAE

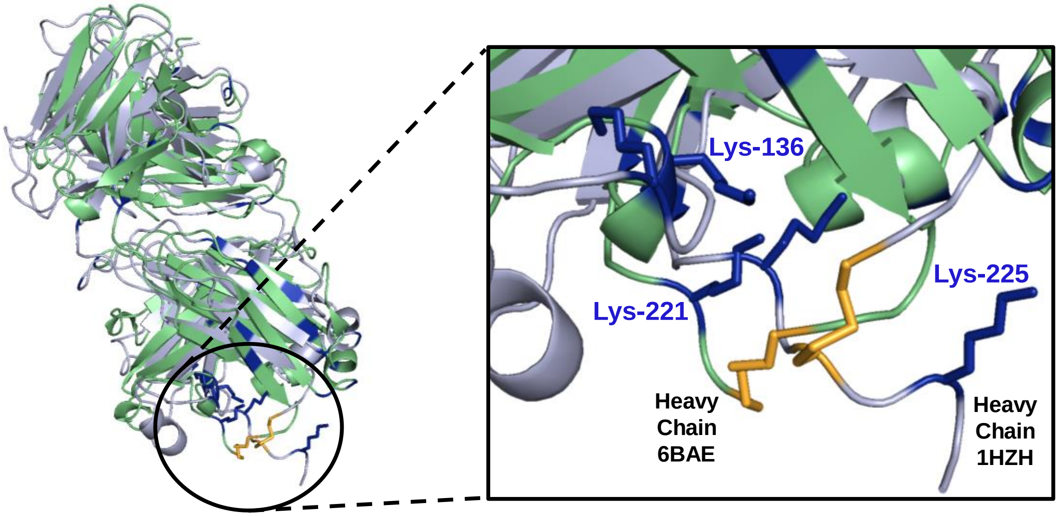

Figure S32 - Overlaid Fab crystal structures, derived from PDB files 1HZH (human IgG1 against HIV-1; shown in gray) and 6BAE (trastuzumab Fab; shown in green); the final four amino acids (i.e. DKTH including Lys-225) are not present in the heavy chain of the PDB file 6BAE.

References

1 M. Slater, M. Snauko, F. Svec and J. M. J. Frechet, *Anal. Chem.*, 2006, **78**, 4969–4975.

2 L. Castañeda, A. Maruani, F. F. Schumacher, E. Miranda, V. Chudasama, K. A. Chester, J. R. Baker, M. E. B. Smith and S. Caddick, *Chem. Commun.*, 2013, **49**, 8187–8189.

3 F. Bryden, A. Maruani, H. Savoie, V. Chudasama, M. E. B. Smith, S. Caddick and R. W. Boyle, *Bioconjugate Chem.*, 2014, **25**, 611–617.

4 M. Morais, J. P. M. Nunes, K. Karu, N. Forte, I. Benni, M. E. B. Smith, S. Caddick, V. Chudasama and J. R. Baker, *Org. Biomol. Chem.*, 2017, **15**, 2947–2952.

5 I. S. Carrico, *Chem. Soc. Rev.*, 2008, **37**, 1423–1431.
